# Pushing Past the Blockade: Advancements in T Cell-Based Cancer Immunotherapies

**DOI:** 10.3389/fimmu.2021.777073

**Published:** 2021-11-18

**Authors:** Jessica Waibl Polania, Emily C. Lerner, Daniel S. Wilkinson, Alexandra Hoyt-Miggelbrink, Peter E. Fecci

**Affiliations:** ^1^ Department of Pathology, Duke University Medical Center, Durham, NC, United States; ^2^ Duke Medical School, Duke University Medical Center, Durham, NC, United States; ^3^ Preston Robert Tisch Brain Tumor Center at Duke, Department of Neurosurgery, Duke University Medical Center, Durham, NC, United States

**Keywords:** immunotherapy, tumor-associated macrophage (TAM), CAR (chimeric antigen receptor) T cells, immune checkpoint inhibition (ICI), tumor microenvironment, immunotherapy resistance, T cell

## Abstract

Successful cancer immunotherapies rely on a replete and functional immune compartment. Within the immune compartment, T cells are often the effector arm of immune-based strategies due to their potent cytotoxic capabilities. However, many tumors have evolved a variety of mechanisms to evade T cell-mediated killing. Thus, while many T cell-based immunotherapies, such as immune checkpoint inhibition (ICI) and chimeric antigen receptor (CAR) T cells, have achieved considerable success in some solid cancers and hematological malignancies, these therapies often fail in solid tumors due to tumor-imposed T cell dysfunctions. These dysfunctional mechanisms broadly include reduced T cell access into and identification of tumors, as well as an overall immunosuppressive tumor microenvironment that elicits T cell exhaustion. Therefore, novel, rational approaches are necessary to overcome the barriers to T cell function elicited by solid tumors. In this review, we will provide an overview of conventional immunotherapeutic strategies and the various barriers to T cell anti-tumor function encountered in solid tumors that lead to resistance. We will also explore a sampling of emerging strategies specifically aimed to bypass these tumor-imposed boundaries to T cell-based immunotherapies.

## 1 Introduction

The advent of immune checkpoint inhibition (ICI) therapies marked the beginning of the cancer immunotherapy resurgence. The remarkable efficacy of ICI therapies against a number of cancer types has prompted enthusiasm for the potential power of immunotherapies in treating both solid and hematologic malignancies. Over the past few decades, advancements in ICI, adoptive immunotherapy, and in our overall understanding of the tumor microenvironment (TME) have led to increasing treatment successes and the FDA approval of numerous immunotherapies, most of which either directly or indirectly impact T cells. Unfortunately, amidst a flood of potential targets and strategies, cancer-induced T cell exhaustion remains a notable obstacle to more widespread efficacy and applicability for T cell-directed immunotherapies.

In this review, we will provide an overview of the barriers and dysfunctions that limit effective anti-tumor T cell responses, and we will discuss emerging therapies that aim to ameliorate these barriers, summarized in **Table 1**. An understanding of novel approaches to address T cell dysfunction will aid the development and implementation of more rationally-designed strategies to combat cancers.

**Table 1:** Emerging therapies to overcome barriers to T cell function.

PROBLEM	SOLUTION	SOURCE
**Exhaustion**		
Metabolic dysfunction	4-1BB, OX40	(1, 2)
Immunosuppressive TAMs	CD40 Agonism	(3)
	CD73, CD39, A2Ar blockade	(4–6)
Alternative ICB	CD161, TIM3, LAG3 inhibition	(7–9)
Adoptive Transfer Exhaustion	Combine with ICI	(10, 11)
	Delete co-inhibitory receptors	(12–14)
	Transient rest	(15)
**Tolerance**		
Tregs	Depleting intratumoral Tregs	(16–19)
	Repolarizing Tregs	(20)
**Infiltration**		
Vascular dysfunction	VEGF/ANG2 dual blockade	(3, 21)
TAM	TAM depletion *via* CCR2 or CSF1R	(22–25)
**Tumor Resistance to T cells**		
Low/No Tumor MHC-I	Recombinant IFNy	(26, 27)
Antigen Heterogeneity - CAR	CAR-secreting BiTE; synNOTCH	(28–30)

Recent work has shed further light on the complexity proffered by the TME, as well as on distinct cell-cell interactions in the TME that limit immune responses to various tumors. Consequently, new strategies have emerged to target the unique immunosuppressive elements within the TME. Along with T cell directed immunotherapies, such as ICI, these strategies are expected to synergistically increase T cell effector function and patient survival.

## 2 Current T Cell-Based Immunotherapeutic Platforms

### 2.1 Immune Checkpoint Inhibition (ICI)

**Figure 1:**
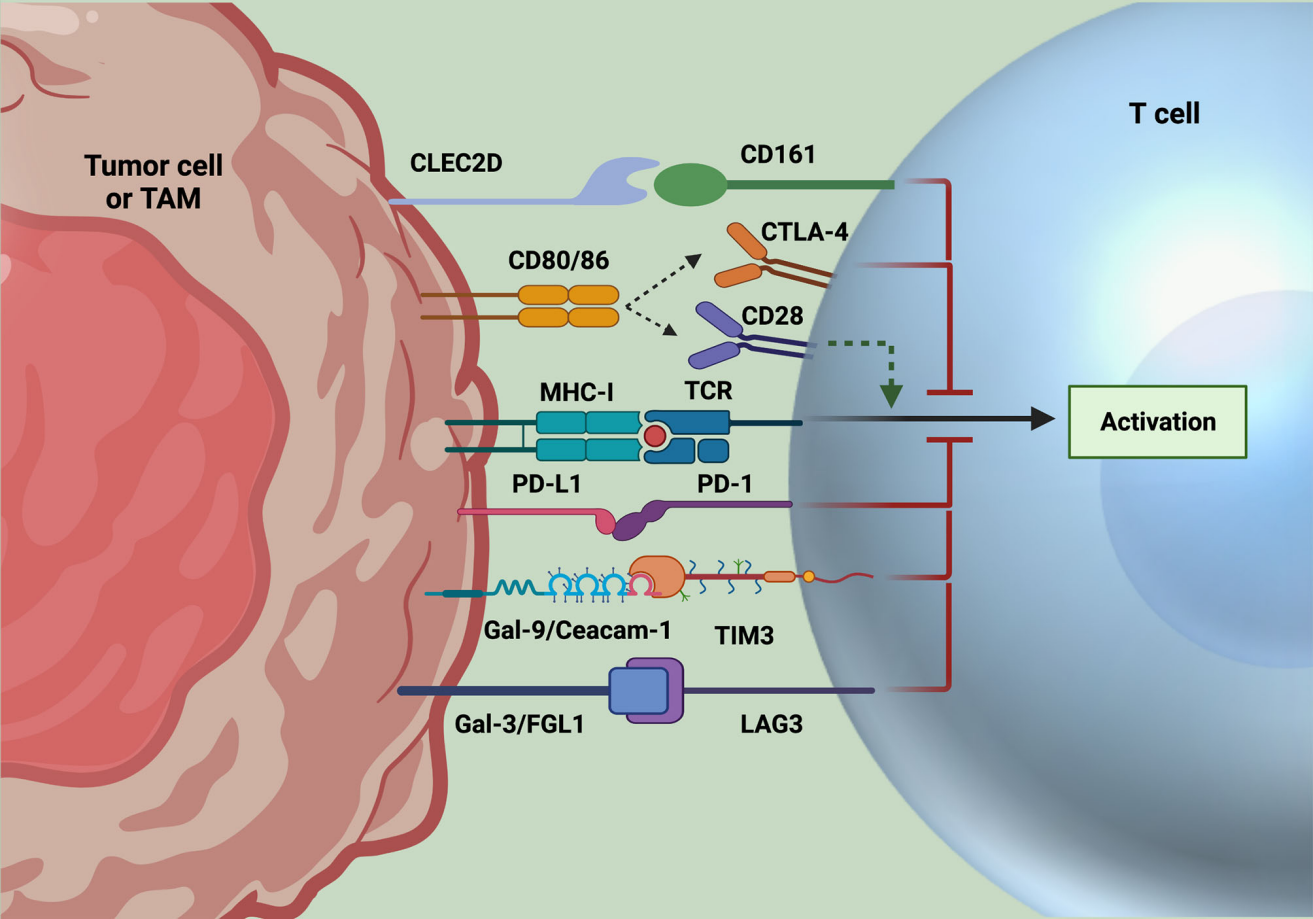
Immune checkpoint inhibition strategies. Classically targeted immune checkpoints include PD1 and CTLA4. However, resistance to these checkpoints alone have led to the discovery of novel targets, including CD161, TIM3, and LAG3, whose ligands are expressed on tumor cells and often TAMs. After binding to their respective ligands, signaling of these immune checkpoints leads to suppression of T cell activation and effector function. Accordingly, these axes have been targeted for ICI, with promising results of increased T cell functionality to date. Created with Biorender.com.

Under homeostatic conditions, the upregulation of cytotoxic T lymphocyte associated protein 4 (CTLA4), programmed cell death protein 1 (PD1), and other immune checkpoints helps to enforce peripheral tolerance by preventing immune-activation by self-antigens, thereby limiting autoimmunity (31). Under acute pathogenic conditions, the induction of immune checkpoints on the surface of activated T cells helps to resolve the immune response after pathogens are cleared, restraining collateral immunopathology. In such homeostatic and infectious circumstances, the expression of immune checkpoints and associated immune restriction is adaptive and limits damage to the host. However, in the case of cancer, the upregulation of immune checkpoints may instead maladaptively limit the anti-tumor immune response and serve an immune-evasive role on behalf of the cancer. Conversely, ICI therapies are intended to release these biological brakes on T cells and allow for prolonged and strengthened immune responses toward malignant cells (**Figure 1**).

In March 2011, the anti-CTLA4 monoclonal antibody (mAb) ipilimumab became the first ICI approved by the FDA, with an initial indication for the treatment of advanced melanoma (32). CTLA4 is expressed on recently activated T cells, where it limits further activation by competing with the costimulatory receptor CD28 for binding with CD80/CD86 on antigen presenting cells (APC). Blocking this interaction with anti-CTLA4 leads to more successful priming and activation of naïve T cells (33). In contrast to CTLA4, PD1 is found on more antigen-experienced T cells. PD1 signaling upon recognition of its ligands, PD-L1 or PD-L2, inhibits T cell receptor (TCR) activation and limits effector activity (34). In 2014, results of the KEYNOTE 001 clinical trial led to FDA approval of the anti-PD1 monoclonal antibody pembrolizumab, the first PD1 ICI for the treatment of advanced melanoma (35, 36).

Drugs targeting CTLA4 and the PD1 pathway have since been applied to a wide range of tumor types including lymphoma, lung cancer, renal cell carcinoma (RCC), head and neck squamous cell carcinoma (HNSCC), bladder cancer, liver cancer, breast cancer, and gastro-esophageal cancer (37). For melanomas, the potential for ICI to create a durable response rate has been demonstrated in multiple clinical trials. The CheckMate 067 study examining nivolumab (anti-PD1) and ipililumab (anti-CTLA4) in combination or as single agents for metastatic melanoma patients reported a 5-year overall survival rate of 52% in the combination group, as compared with historical 5-year survival rates under 20% (38, 39). The response rates for many other tumor types, however, have not been as strong, due to tumor intrinsic and extrinsic factors, such as heterogeneity and mutational burden, and the tumor microenvironment, respectively (40).

A 2020 cross sectional study estimated that approximately 38% of cancer patients in the USA were eligible for immunotherapies in 2018, and of them, less than 12% of patients responded to therapy (41, 42). Resistance to ICI therapy was evident in the initial KEYNOTE 001 study, where within the cohort of advanced melanoma patients, only 16% achieved a complete response (CR), with a median follow-up time of 2 years (35). Factors contributing to ICI therapy resistance include poor T cell access to and function within the tumor, which are often due to the presence of immunosuppressive cells and soluble factors in the TME, as well as tumor cell intrinsic factors including low tumor mutational burden and high heterogeneity (43). Therefore, novel strategies that overcome these barriers must be developed to expand the repertoire of cancers upon which ICI is effective.

### 2.2 CAR T Cells

Chimeric antigen receptor (CAR) T cells represent a subclass of adoptive immunotherapy. CAR T cells are most often CD8 T cells that have been genetically modified to express an extracellular antigen-targeting moiety, typically an antibody single-chain variable fragment (scFv) in tandem with intracellular T cell activating domains (44). These intracellular domains consist of the CD3ζ chain, along with CD28 and/or 4-1BB co-stimulatory components. Thus, T cells are redirected to recognize a cell-surface antigen of choice in a major histocompatibility complex (MHC)-independent manner. This MHC-independency provides CAR T cells a significant advantage over conventional TCR-bearing T cell adoptive therapies, allowing CAR T cells to bypass the low immunogenicity adapted by many tumor types.

**Figure 2:**
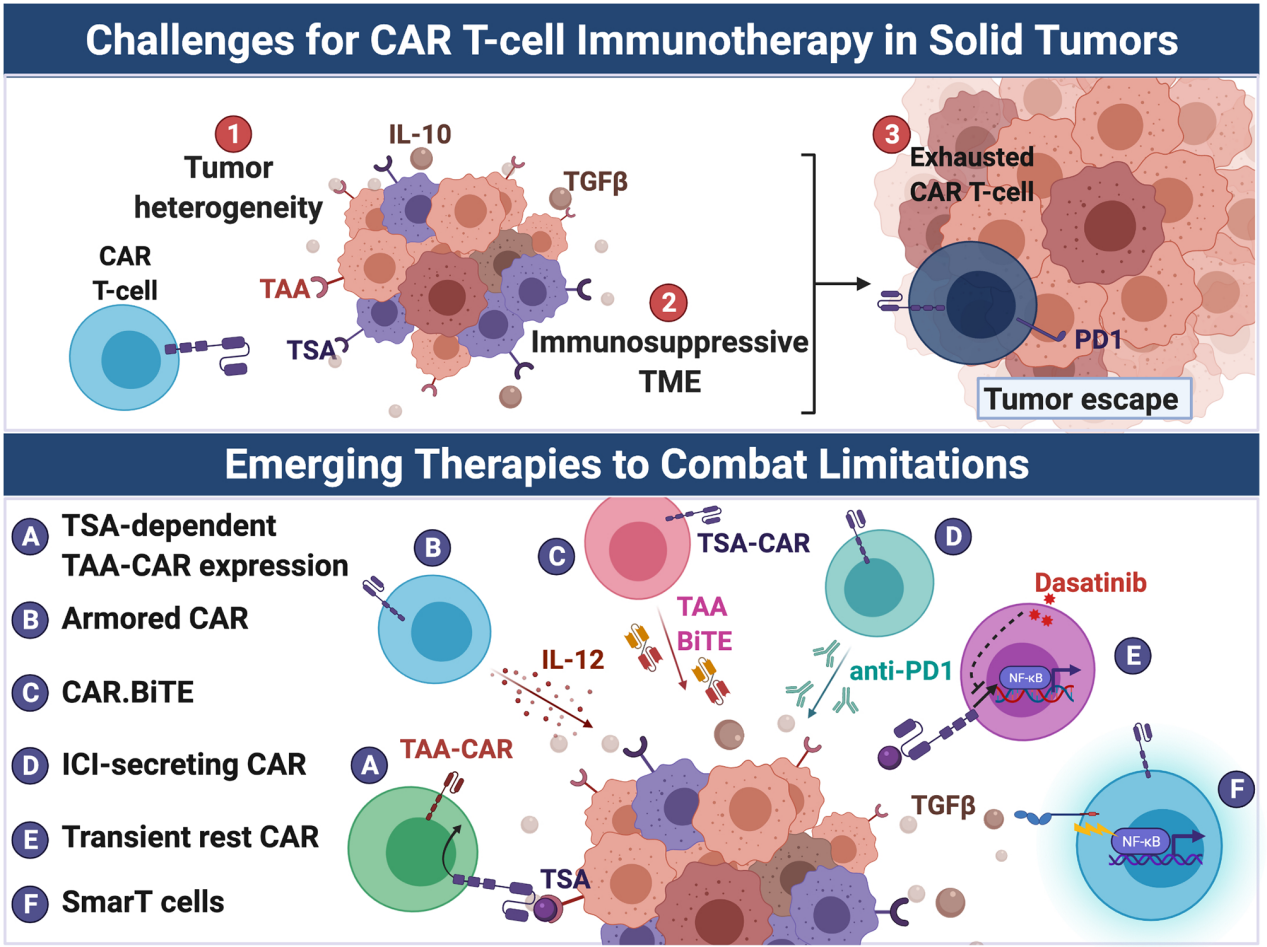
Emerging therapies to overcome challenges to CAR-T cell immunotherapy in solid tumors. CAR T cell therapeutic efficacy has been greatly hindered in solid tumors due to (1) tumor heterogeneity, (2) the immunosuppressive TME, and (3) induction of CAR T cell exhaustion. Several strategies have been developed to overcome these barriers. Tumor heterogeneity results in antigen escape from targeted CAR therapy by antigen-negative tumor cells. To overcome the limitation of tumor heterogeneity, CAR T cells have been engineered to: **(A)** target multiple antigens including more ubuquitously-expressed TAAs but only after recognition of a TSA, and, similarly, **(B)** secrete cytokines such as IL-12 that promote endogenous anti-tumor immunity, **(C)** secrete BiTEs that target TAAs, thus resulting in intratumoral redirection of endogenous T cells to tumor cells. The immunosuppressive milieu (e.g. suppressive cytokines such as TGF-β) of the TME represses maximal CAR T cell activation. Additionally, solid tumors induce T cell exhaustion of CAR T cells through chronic antigen stimulation and tonic CAR signaling. To counter exhaustion, CAR T cells were: **(D)** engineered to secreted anti-PD-1 and **(E)** treated with dasatinib to prevent chronic CAR signaling and enhance CAR activation upon encounter of tumor. **(F)** To evade the immunosuppressive TME, SmarT CAR T cells were developed by fusing extracellular TGF-β and IL-4 receptors to intracellular co-stimulatory signaling domains, thus converting the action of TGF-β and IL-4 from inhibitory to stimulatory. Created with Biorender.com.

CAR T cells have achieved success in certain hematological malignancies, including relapsed acute lymphoblastic leukemia (ALL) and refractory chronic lymphocytic leukemia (CLL), resulting in recent FDA approval of two CD19-targeting CAR products (45). However, similar success in solid tumors has yet to come to fruition. These failures are due jointly to solid tumor heterogeneity, the immunosuppressive TME, and CAR T cell exhaustion (**Figure 2**). To develop rationally-designed therapies that overcome current modes of resistance, it is necessary to first understand the factors limiting their efficacy.

## 3 Factors Limiting The Success Of Immunotherapy

### 3.1 Lack of T Cell Access

Conventional anti-tumor cytotoxic T cells function by recognizing processed antigen in the context of MHCI on the surface of tumor cells, while CAR T cells directly recognize native antigen on the surface of tumor cells. Before recognizing tumor cells, they must gain access to the tumor, overcoming tumor-imposed boundaries.

#### 3.1.1 Disorganized Neovasculature and Stromal Barriers

Among solid tumors, there is stark variability in TME composition and immune cell infiltration. Traditionally, “cold” and “hot” have been used to describe cancers with high and low levels of infiltration, respectively. Cold tumors, such as brain tumors and pancreatic ductal adenocarcinoma (PDAC), often consist of a high macrophage to T cell ratio (46–48). Notably, T cells in these tumors display decreased functional capacity, in both humans and mice (49, 50). In contrast, hot tumors, including skin melanoma, lung adenocarcinoma, and head and neck squamous cell carcinoma (HNSCC) have much higher T cell to macrophage ratios. This has been associated with increased responsiveness to immunotherapy (51). Factors that contribute to the degree of T cell infiltration in cancers include blood vessel permeability and organization, as well as macrophage phenotypes and composition. Immune cell extravasation and infiltration into the tumor is highly regulated by expression of adhesion molecules and cohesiveness of endothelial cells (i.e., tight junctions). Additionally, expression of adequate adhesion molecules, and permeability are highly dependent on location (52). For example, blood vessels of the liver exhibit discontinuous endothelial cells and therefore allow for cellular infiltration. In contrast, blood vessels in the brain are characterized by tight junctions and astrocyte foot processes (known as the blood-brain barrier), which greatly restrict immune cell extravasation under normal conditions (53, 54). Tumors alter the surrounding vasculature to increase nutrient entry through the secretion of endothelial growth factors, such as vascular endothelial growth factor (VEGF) and angiopoietin 2 (ANG2). These new blood vessels are often disorganized and lack the expression of molecules required for extravasation, thereby limiting T cell trafficking (55).

Once outside of blood vessels, infiltrating T cells face additional barriers that prevent further infiltration into the tumor, such as dense extracellular matrix and tumor-associated macrophages (TAM) (46). Increased density of macrophages in the surrounding tumor stroma has been shown to limit T cell infiltration into the tumor, largely due to their roles in tissue remodeling and recruitment of cancer associated fibroblasts (56, 57). In PDAC, for example, macrophage production of granulin promotes the accumulation of myofibroblasts (58). Furthermore, macrophage-derived transforming growth factor-beta (TGF-β) has been shown to limit T cell entry in metastatic urothelial and colon cancers (59–61). For these reasons, TAMs have become one of the foremost targets of immunotherapy in recent years. Importantly, combinatorial approaches are often successful, due to their ability to relieve more than one aspect of T cell suppression.

Still, in some cases, T cell infiltration alone does not correlate with improved prognosis or response to immunotherapy, suggesting that intratumoral entry alone is not sufficient to elicit a successful anti-tumor response (62, 63). High heterogeneity and low tumor mutational burden limit the ability of infiltrating T cells to carry out their anti-tumor functions. Furthermore, immunosuppressive cytokines, chronic antigen exposure, and inhibitory signaling can all lead to various modes and degrees of T cell dysfunction within the tumor.

#### 3.1.2 Tumor Antigens and Mutational Burden

As mentioned previously, T cells recognize antigen on the surface of tumor cells, either in the context of MHCI for endogenous CD8 T cells or natively by CAR T cells. Tumor cells have adapted mechanisms to evade recognition by CD8 T cells by downregulating MHCI. Mutations leading to decreased or absent MHCI expression, most notably through the loss of functional beta-2 microglobulin expression, constitute a common mechanism by which tumors evade immune detection, rendering them immunologically cold (64–67).

Even with preserved MHCI expression, tumors can evade immune detection by reducing expression of tumor specific antigens (TSA), often termed neoantigens. Tumors acquire mutations as they proliferate, these mutations are reflected in the heterogenous set of antigens expressed on surface MHCI. The accumulation of non-synonymous mutations leads to the expression of altered peptide sequences on cell surface MHC-I, known as neoantigens, that are recognized as non self-antigens by immune cells (64, 68–70). Tumors with high neoantigen expression are therefore more immunogenic and postulated to be the target of CD8 T cells in response to checkpoint inhibition (70, 71). Tumors cells can also present tumor associated antigens (TAAs), which are normal self-antigens that are expressed on both healthy cells and tumor cells, although the level of expression may be higher in tumor cells (68, 72, 73). Unlike TAAs, neoantigens are not recognized as self, and therefore not subject to the same central or peripheral modes of tolerance as are TAAs (68, 70).

Higher mutational burden is associated with increased frequency of neoantigens. As such, tumors with high mutational burden, and therefore, high neoantigen expression are more responsive to T cell directed immunotherapies. Accordingly, the FDA approved indications for ICI drugs have been expanded to include tumors with high mutational burden, including tumors with evidence of high microsatellite instability (MSI-H) or mismatch repair (MMR) deficiencies (74). High neoantigen expression is not sufficient to produce an effective anti-tumor response, however, as T cells specific to the diverse array neoantigen peptides must also be present. In order to mount an effective anti-tumor CD8+ T cell response in the setting of an immunogenic tumor, TILs must be both active effector cells and have sufficient diversity of T cell receptors to provide specificity for the heterogenous antigens displayed by tumors. Several recent clinical studies on cohorts of patients with renal cell carcinoma, NSCLC, and melanoma have provided evidence for the significance of high T cell receptor diversity in predicting improved response to checkpoint blockade therapy (75–77).

#### 3.1.3 Tumor Heterogeneity

Increased tumor mutational burden can be beneficial when it results in increased tumor neoantigen expression, however, heterogeneity of antigen expression between tumor cells can be problematic for therapies that target a specific tumor antigen, such as CAR T cells.

Tumor antigen expression is heterogeneous in that tumor cells often differ in their antigen expression profiles, both from patient to patient and within the same tumor. Thus, antigen-targeted therapies, such as CAR T cells, are typically only successful when all tumor cells within a tumor express the targeted antigen. Unfortunately, unlike their “liquid” counterparts, solid tumors do not tend to afford such universal antigenic expression. For example, EGFRvIII is one of the only and most highly expressed TSA characterized in GBM. However only approximately 30-50% of GBM tumors express EGFRvIII, and only about 30-50% of tumor cells in “EGFRvIII^+^” tumors express the antigen (78). Our pre-clinical and clinical experiences with CAR T cells reveal that tumors possessing as few as 5-10% EGFRvIII-negative cells will easily escape EGFRvIII-targeted CARs (79). Moreover, antigen escape is documented in a variety of additional cancer types following CAR treatment, including hematologic malignancies (80, 81). The driver of intratumoral heterogeneity seems to be the genomic instability that is characteristic of tumor cells, with different individual cells acquiring different mutations (82–84). This branching evolution, as opposed to a more linear evolution, results in a tumor consisting of subclones with varied antigen profiles (16, 85, 86). Tumor heterogeneity is a major barrier to successful CAR T cell therapy in solid tumors, and strategies to overcome this hurdle will be discussed subsequently.

### 3.2 Suppressive TME

**Figure 3:**
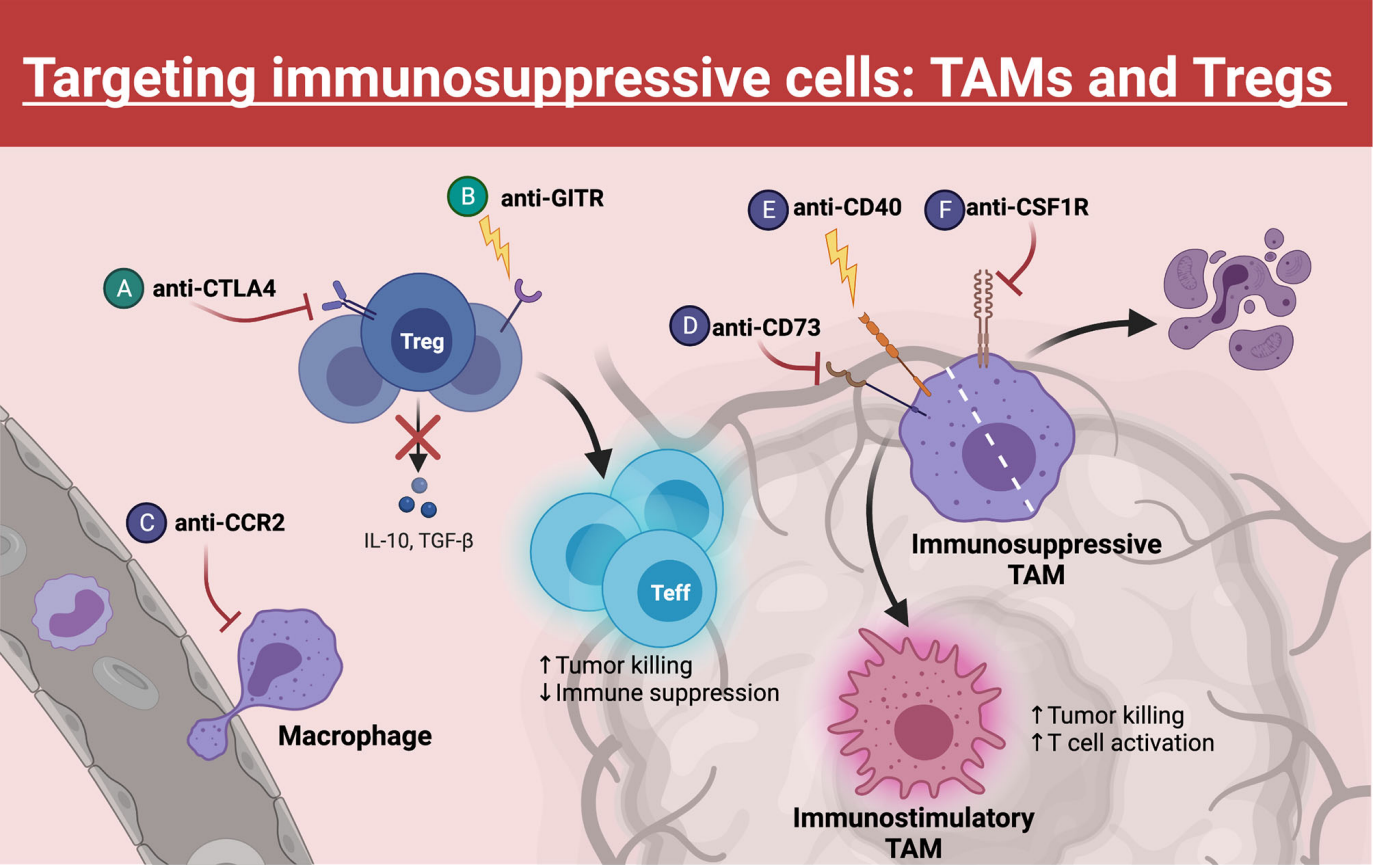
Targeting TAMs and Tregs. Within the TME, Tregs and TAMs represent major barriers to effective anti-tumor T cell function. Many emerging therapies have been developed to target these populations, which often synergize with immune checkpoint blockade. **(A)** Blocking CTLA4 on Tregs decreases their immunosuppressive function, whereas **(B)** GITR agonism can destabilize Tregs, causing these Tregs to gain effector function. **(C)** TAM trafficking to the tumor can be inhibited through blockade of surface CCR2. **(D, E)** Repolarizing TAMs from an immunosuppressive phenotype to an immunostimulatory phenotype can be achieved through CD73 inhibition or CD40 agonism. **(F)** Another strategy to eliminate TAMs within the TME is blocking of CSF1R, a critical growth receptor on TAMs, which leads to cell death. Created with Biorender.com.

Once T cells have gained access to the tumor, T cells must face a complex milieu of cells and soluble factors in and around a tumor that is generally presumed to be immunosuppressive. Major contributors to such immunosuppression are tumor-recruited anti-inflammatory cell populations, most frequently TAMs and regulatory T cells (Treg). These cell populations can repress tumoricidal immune responses within the TME through tissue remodeling (creating hypoxic environments) and inhibitory interactions, hindering T cell effector capacity. Accordingly, emerging therapies often target these populations in efforts to better license T cell-based therapies (**Figure 3** **).**

#### 3.2.1 Suppressive TAMs and Tregs

Macrophages represent a multifunctional class of immune cells that can operate broadly as either pro-inflammatory or anti-inflammatory mediators. In most tumors, TAMs differentiate from monocytes that migrate to the tumor site in response to inflammatory signals secreted by tumor cells. Notably, gliomas and pancreatic cancers also contain resident macrophages of embryonic yolk-sac origins that may play differential roles in tumor progression (87–89). In murine PDAC models, monocyte-derived TAMs participated in antigen presentation and shaping the immune response, whereas resident macrophages with a pro-fibrotic transcriptional profile were more involved in producing and remodeling the extracellular matrix (89). Interestingly, microglia within a murine model of GBM were enriched for expression of inflammatory cytokines, whereas monocyte-derived TAMs were enriched for wound healing-associated chemokines and expression of Aryl-hydrocarbon receptor (Ahr), a transcription factors associated with immune suppression (87, 88, 90). Within the TME, pro-tumor TAMs dominate and can promote tumor growth through secretion of growth factors and, in part, by dampening effector T cell function through secretion of anti-inflammatory cytokines, TGF-β and interleukin (IL)-10, as well as expression of immune modulators, such as PD-L1 (87, 91, 92). Additionally, macrophages can promote angiogenesis and tumor cell metastasis (93, 94). Thus, these immunosuppressive TAMs negatively impact T cell targeting immunotherapies and must be countered to improve T cell-based therapeutic strategies.

The importance of Tregs under homeostatic conditions is illustrated by the disease Immune Dysregulation, Polyendocrinopathy, Enteropathy, X-linked (IPEX). Patients with IPEX have mutations in the Treg master transcription factor *FOXP3* gene, resulting in the dysfunctional development of Tregs and leading to fatal multi-organ autoimmunity within the first two years of life (95, 96). Moreover, Treg deficiencies have been identified in patients suffering from various autoimmune disorders including multiple sclerosis (MS) (97), mysasthenia gravis (98), and type 1 diabetes (T1D) (99). Unsurprisingly, many cancers have usurped the suppressive nature of Tregs to thwart effective antitumor immunity. Tregs are often found in high proportions in the tumor microenvironment of solid cancers, where they act to support an immunosuppressive tumor microenvironment (100–105). Tregs utilize a variety of mechanisms to suppress effector T cell activation. These mechanisms include modulating antigen presenting cells (APCs) function through competitive blockade of CD80:CD28 costimulation with CTLA4 (106), secreting immunosuppressive soluble mediators like TGF-β and IL-10 (107, 108), and depleting local IL-2 pools due to their constitutively high CD25 (IL-2Rα) expression (109), among many others (110). Consequently, targeting Tregs to enhance antitumor immunity or to augment the efficacy of immunotherapies signifies a rational and promising strategy.

#### 3.2.2 Hypoxia

The TME often contains hypoxic regions, resulting from locally disorganized vasculature and uncontrolled tumor cell proliferation. Hypoxia has been documented as a contributor of T cell exhaustion, due to its potential negative impacts on T cell metabolism (111, 112). Interestingly, T cells have evolutionarily acquired a mechanism to overcome this barrier through upregulation of hypoxia-responsive factors. Hypoxia inducible factor 1 subunit alpha (HIF-1α) is a transcription factor that is rapidly degraded under normoxic conditions but becomes stabilized and activated by hypoxic conditions (113, 114). When activated, HIF-1α aids in reprogramming cellular metabolism to function in hypoxic conditions by triggering a switch from oxidative to glycolytic metabolism (113, 115–117).

### 3.3 T Cell Exhaustion

T cell exhaustion is one of the most studied forms of T cell dysfunction due to its well-documented role in limiting the adaptive anti-tumor response (118). During many normal immune responses to chronic pathogens (especially chronic viral infection), T cell exhaustion can evolve as a programmed host-adaptive stalemate between the immune system and the host, in order to minimize immune-related collateral damage to normal tissues. Unfortunately, in the context of cancer, T cell exhaustion can be co-opted to foster tumor growth and contribute to cancer immune evasion. T cell exhaustion was initially described in the context of chronic lymphocytic choriomeningitis (LCMV) infection as a progressive and stereotyped hierarchical loss of memory and effector T cell function that is maintained in an antigen-dependent manner (119). Subsequently, years of research have led to the designation of T cell exhaustion as a separate T cell differentiation state with a unique transcriptional and epigenetic program (120, 121). The causes of T cell exhaustion are multifactorial and continue to be an active area of investigation. It was recently shown in a murine model of melanoma that metabolic function, and specifically mitochondrial fitness, is directly linked to the development of an exhausted phenotype (111).

T cell exhaustion is most often characterized by high expression of coinhibitory receptors, such as PD1 and CTLA4. However, alternative coinhibitory receptors have also been identified, including T cell immunoglobulin domain and mucin domain 3 (TIM3), and lymphocyte-activation gene 3 (LAG3). Increased expression of these molecules by T cells has been shown to limit the efficacy of immunotherapy in cancer (118). An extensive body of work now exists that details the current framework of CD8 T cell exhaustion in both chronic infection and cancer [reviewed in (122)]. Less is understood about CD4 T cell exhaustion, although CD4 T cell exhaustion is also likely important in the context of chronic infection and tumor immunology [reviewed in (123)].

Importantly, two major subsets of T cell exhaustion have recently been identified, with these termed “progenitor” and “terminal” exhaustion. Progenitor exhaustion refers to a stem-like population of intermediate PD1 expressing (PD1^int^) T cells with proliferative potential and long-term self-renewal capabilities (124–126). This population has been shown to express memory-associated markers, such as TCF1, IL7R, and CXCR5 (127–129). Terminal exhaustion is characterized by high expression of PD1, TIM3 and other coinhibitory receptors, increased apoptotic signaling, and a cytotoxic program (129, 130). The importance of these subsets is underscored by their dictating differential responses to ICI. As early as 2008, it was shown that a PD1^int^ population could proliferate upon PD-L1 blockade and mediate control of a chronic viral infection (131). Further analysis of this PD1^int^ population demonstrated a 30-fold increase in its transition toward terminal exhaustion following PD-L1 blockade compared to the untreated controls (124).

From these studies, a model was proposed where progenitor exhausted T cells act as exhaustion “stem cells” during chronic infection that undergo slow self-renewal while also giving rise to the effector terminal exhaustion population (124, 129). Notably, this paradigm has been supported in models of melanoma, colorectal cancer, glioblastoma, and others (50, 130, 132). As an example, Miller et al. used single cell RNA sequencing (scRNAseq) to identify the stem-like progenitor exhaustion population and the more effector-like terminal exhaustion population in a murine model of melanoma. Researchers noted that the progenitor exhausted population displayed greater polyfunctionality (TNFα^+^IFNγ^+^) and the ability to persist without antigen, whereas the terminally exhausted population had superior cytotoxicity (granzyme B (Gzmb)^+^ IFNγ^+^) and reduced long-term survival. In adoptive transfer experiments, groups with transferred progenitor exhausted cells, but not transferred terminally exhausted cells, had enhanced tumor control and retained responsiveness to PD1 blockade. Additionally, in human advanced melanoma samples, higher frequency of progenitor exhaustion correlated with improved duration of response to ICI. The authors concluded that strategies to stimulate the progenitor exhausted population more specifically are likely to improve the efficacy of ICI. Altogether, an increased understanding of T cell exhaustion will continue to guide elucidation of novel targets and newer angles on immunotherapy, which will be discussed in further detail below.

## 4 Emerging Advances That Overcome Barriers To T Cell-Based Immunotherapy

The barriers described above limit the effectiveness of T cell-based immunotherapies. Novel, rational approaches that aim to overcome current limitations of immunotherapies are discussed here.

### 4.1 Enhancing T Cell Access

#### 4.1.1 Increasing T Cell Infiltration

The angiogenic signal protein VEGF is upregulated by HIF-1α activation and plays an important role in the dysfunctional vasculature of the TME. At steady state, the development of new blood vessels is necessary for physiological processes, such as embryogenesis, organogenesis, and wound healing. This is a highly regulated process to ensure sufficient nutrients are reaching tissues without uncontrolled growth (133). Sustained angiogenesis has been recognized as a hallmark of cancer for decades. Early studies identified the requirement for angiogenesis for sustained tumor growth after nearby nutrients had been exhausted. These findings, coupled with the discovery of VEGF overexpression in tumors and the tumor-suppressive effects of VEGF inhibition (133), cemented the importance of angiogenesis in the growing cancer field. More recently, tumor-induced angiogenesis has been shown to produce an abnormal vasculature that may limit lymphocyte infiltration as a result of altered expression of adhesion molecules, chemokines, and cytokines (55).

Traditionally, anti-angiogenics, such as anti-VEGF or anti-ANG2, aim to prevent tumor neovascularization to limit the entry of necessary nutrients and immunosuppressive cells. Recent studies have shown that this strategy may also normalize previously abnormal blood vessels, improving the extravasation of CD8 T cells into the tumor site (3, 55). To date, anti-VEGF-A antibodies have been the most common anti-angiogenic treatment modality. These have been paired with standard of care treatments and ICI to extend PFS (134). However, many cancers demonstrate resistance to anti-VEGF-A treatment alone, including glioblastoma (21). It is hypothesized that ANG2 production mediates this resistance. Accordingly, dual blockade of VEGFA and ANG2 has demonstrated superior preclinical results through increased T cell tumor infiltration and myeloid repolarization (3). In this study, researchers observed improved perfusion and reduced leakiness of blood vessels within treated tumors. The normalized vasculature was associated with increased CD8 T cells infiltration. This was augmented by combination with anti-CD40 agonist, which further promoted intratumoral infiltration of CD8 T cells and tumor eradication in several syngeneic murine tumor models. Macrophages in this model were repolarized from an anti-inflammatory signature towards a pro-inflammatory one, highlighting potential synergy between strategies to normalize tumor vasculature and those to enhance T cell function.

While blocking VEGF-A expression is one approach, Song et al. instead demonstrated benefits to combining ICI with forced ectopic expression of VEGF-C in a syngeneic model of murine glioblastoma (135). The authors identified enhanced CD8 T cell priming following imposed increases to VEGF-C-driven lymphatic drainage of the TME. These findings reveal the importance of lymphatic drainage for guiding an effective anti-tumor T cell response, as well as challenge the dogma that inhibiting angiogenic factors is the correct strategy. Interestingly, VEGF-C is often a biomarker for metastasis in peripheral cancers (136, 137), highlighting the importance of understanding the nuanced roles of the various angiogenic factors.

#### 4.1.2 Combatting Tumor Heterogeneity

One approach taken to combat such intratumoral heterogeneity is to attempt to elicit “bystander” immune responses and epitope spreading. An example is the recent production of IL-12-secreting CARs, often termed “Armored CARs”, which are aimed at amplifying neighboring polyclonal Th1 immune responses within the TME. Local release of IL-12 in the TME is thought to promote T cell priming and activation by DCs, thereby enhancing the endogenous anti-tumor response (138). However, due to the potential toxicity of IL-12 constitutively secreted by CARs (139), CARs that only secrete IL-12 locally within the TME upon CAR engagement are currently being developed. Liu, et al. recently generated a Glypican-3-targeting CAR that contained IL-12 under the control of an NFAT-dependent promoter (140). Thus, IL-12 was only secreted upon CAR activation. In a preclinical model of hepatocellular carcinoma (HCC), these inducible IL-12 Glypican-3 CARs significantly improved survival of mice compared to non-IL-12-secreting Glypican-3 CARs. Unfortunately, this study did not measure *in vivo* efficacy of inducible IL-12 CARs in the context of antigen-heterogeneous tumors. However, the superior anti-tumor effect of inducible IL-12-secreting *versus* non-IL-12-secreting CARs provides evidence supporting pursuance of this strategy.

Multi-antigen-targeting CAR-based T cell therapies are another way to combat tumor heterogeneity. Unfortunately, many of the most highly expressed antigen targets are often only tumor-associated, not tumor-specific. If these TAAs are to be targeted, extra caution must be taken to avoid on-target, off-tumor toxicities. Choi, et al. took a creative approach to combat tumor heterogeneity in gliomas that have variable expression of EGFRvIII and wildtype (WT) EGFR (28). WT EGFR is a TAA overexpressed in many gliomas and absent on normal CNS tissue, but it is expressed on normal peripheral tissues. Therefore, a WT EGFR-targeted therapy must be delivered locally to the tumor to prevent any on-target, off-tumor toxicities. Choi, et al. generated an EGFRvIII-targeting CAR that secretes a WT EGFR-targeting bi-specific T cell engager (BiTE). These CART.BiTEs were shown to be effective when administered intracranially to mice bearing brain tumors, with the CART.BiTEs directly killing EGFRvIII^+^ tumor cells, while also redirecting endogenous T cells to kill WT EGFR^+^ tumor cells. Another recently reported approach utilized a synthetic Notch (synNotch) system to control surface CAR expression (29, 30). In this system, surface expression of a TAA-specific CAR construct is dependent upon signaling through a constitutively expressed TSA-specific CAR on the same T cell. Thus, the TAA CAR construct remains hidden until the CAR T cell has reached the tumor and encountered the relevant TSA. This approach was preclinically shown to be effective in models of GBM, mesothelioma, and ovarian cancer, suggesting broad applicability for this platform.

### 4.2 Overcoming TME-Mediated Immune Suppression

#### 4.2.1 Targeting TAMs

To target TAMs, one strategy involves stimulating CD40 on TAMs with an anti-CD40 agonist, which can activate and polarize them towards a pro-inflammatory phenotype (increased costimulatory and MHC molecule expression). CD40 is a tumor necrosis factor (TNF)-receptor superfamily member that is primarily expressed on APCs, such as macrophages, DCs, B cells, and monocytes (141). When bound to its ligand, CD40L, CD40 induces activation of TAMs and other APCs, leading to upregulation of costimulatory and MHC molecules. Notably, there is strong pre-clinical evidence that tumor regression can follow treatment with agonist CD40-targeting antibody (3, 141). This strategy is believed to enhance T cell priming by TAMs (and other APCs). Interestingly, chemotherapy, when followed by CD40 agonism (but not vice versa) in murine solid tumor models, has elicited effective T cell responses, tumor clearance, and T cell memory. This study suggested that CD40 agonism may lead to better priming of T cells by APCs when these APCs are loaded with tumor antigens recovered from dying tumor cells (142). These results have informed several phase I clinical trials that are testing CD40 agonism in combination with chemotherapy (NCT01103635, NCT02705196, NCT03214250).

Additionally, ongoing efforts are focused on combining the immunostimulatory effects of anti-CD40 with other therapies, such as anti-PD1 or anti-angiogenics, to capitalize on the enhanced T cell activation potential of repolarized myeloid cells. In an ongoing phase 1b clinical trial, examining CD40 agonism in combination with chemotherapy ± ICI (nivolumab), in patients with untreated PDAC demonstrated partial responses in 14/24 patients, and stable disease in 8/24 (NCT03214250) (143). Another phase 1 clinical trial demonstrated an overall response rate (ORR) of 27.3%, which included 2 complete responses and 4 partial responses, in 22 metastatic melanoma patients treated with CD40 agonism (selicrelumab) in combination with anti-CTLA4 (tremilimumab) (NCT01103635). Notably, 9 patients had long-term survival of over 3 years (144).

**Figure 4:**
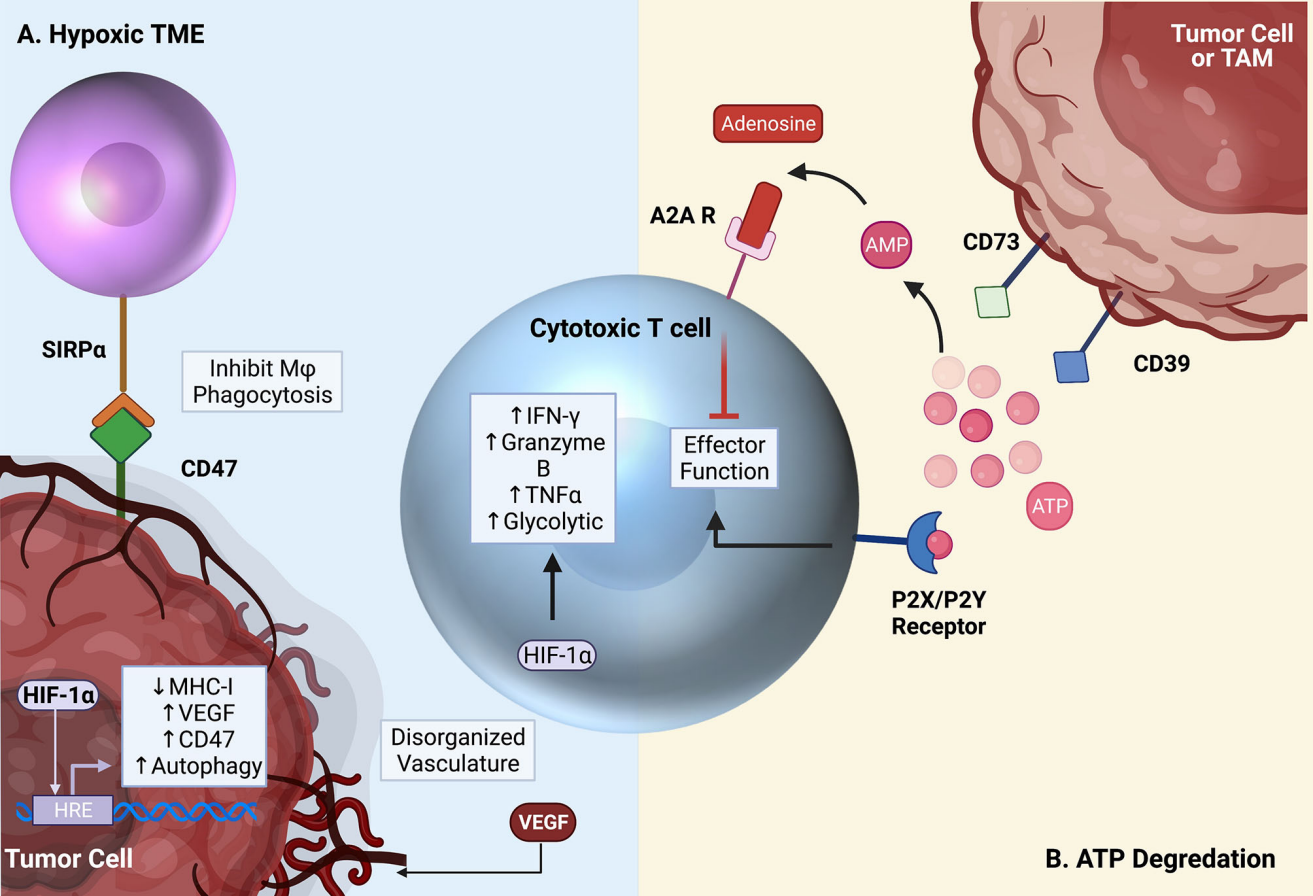
Critical pathways targeted by emerging therapies. **(A)** The TME is characterized by hypoxia and low glucose. Increased VEGF expression by tumor cells and local immune cells leads to the creation of disorganized vasculature, ultimately worsening the metabolic environment. HIF-1α leads to the activation of several pro-tumor pathways, but also contributes to supporting T cell effector function in the hypoxic TME. **(B)** Free ATP in the TME activates T cells through the P2X/P2Y receptors. In the TME, however, free ATP is converted to immunosuppressive adenosine by surface CD39 and CD73 expression on tumor cells and TAMs. Adenosine then binds to the A2A receptor on T cells, inhibiting effector function. Created with Biorender.com.

Another strategy targeting TAMs involves inhibiting CD73. CD73, along with CD39, are rate limiting ectonucleotidase enzymes involved in the extracellular degradation pathway of ATP to adenosine (**Figure 4**) (4, 145–147). Both ATP and adenosine serve as immunologic signaling molecules within the TME, though with opposite effects. ATP is released into the extracellular compartment by dead and dying tumor cells and serves as a pro-inflammatory signal through binding to the P2X/P2Y receptors on T cells (147). Free extracellular ATP can also be converted to adenosine monophosphate (AMP) by CD39, and AMP is subsequently converted to adenosine by CD73 (4, 145–147).

In contrast to ATP, extracellular adenosine has immuno-suppressive effects. Adenosine binds to the A2A receptor on cytotoxic T cells resulting in their death (148, 149). Under homeostatic conditions, this process controls unwarranted inflammation in response to the continuous death and turnover of normal cells. In the context of cancer, apoptosis of anti-tumor effector T cells is clearly detrimental to the desired anti-tumor immune response. A major source of adenosine within the TME derives from CD73 expression on macrophages and other myeloid cell populations. This depot of intra-tumoral adenosine in turn can hinder T cell-based immunotherapies (5). Therefore, inhibiting CD73, especially on TAMs, is of great interest. Notably, CD73 was highly identified on TAMs in glioblastoma through an effort to immune profile several cancers (6). Goswami et al. identified a CD73^hi^ TAM population that persisted following anti-PD1 treatment in glioblastoma patients. Furthermore, this group found an enhancement of ICI efficacy in CD73 knockout (CD73KO) mice. In these mice, intracranial tumor growth was impeded, and prolonged survival was observed. Interestingly, researchers observed no change in T cell subsets within CD73KO mice alone, but when ICB was administered, increased T cell infiltration and activation resulted. Additionally, repolarization of macrophages from anti-inflammatory (CD206^+^) to pro-inflammatory (iNOS^+^) phenotypes was observed. Importantly, CD73 expression is not restricted to TAMs. In fact, many tumor cells express high levels of both CD39 and CD73, usurping the immunosuppressive effects of this pathway to further inhibit antitumor immunity (146, 150). In gastric cancers, high tumor CD73 expression has been associated with poor overall survival and advanced clinical stage. These findings support the use of strategies aimed at countering immune-suppressive mechanisms employed by TAMs as a potentially effective immunotherapeutic adjunct. Phase I clinical trials are currently underway in PDAC (NCT03611556), breast cancer (NCT03742102), and many other solid tumors (NCT03454451) to evaluate the safety and efficacy of CD73 blockade in combination with PD1 axis inhibitors (anti-PD1, anti-PDL1) (151).

Strategies to remove TAMs from the TME have also been investigated. Depleting macrophages from the tumor site can be accomplished by inhibiting their trafficking or entry into the TME, or by blocking macrophage survival signals to instigate their death. Macrophage trafficking can be suppressed by targeting the CCL2/CCR2 axis. CCL2 is an important chemokine involved in monocyte recruitment. It is highly expressed by many cancer types, making it an attractive target for limiting macrophage infiltration into the TME. Overexpression of CCL2 has been documented in patients with lung adenocarcinoma, breast cancer, and hepatocellular carcinoma (152–154). Disappointingly, limited benefit has been observed in clinical trials with therapies targeting the CCL2-CCR2 axis alone. However, there is strong evidence from several preclinical tumor models supporting the combination of CCR2 inhibition and ICI (155). For instance, Flores-Toro et al. found synergy between a CCR2 antagonist and anti-PD1 treatment. In this study, median survival in a murine model of glioma was extended by 46% (35 d *vs*. 24 d), with such survival benefit correlating with elevated expression of IFN-γ by CD8 T cells (22).

In contrast to preventing macrophage tumor infiltration, efforts to deplete them entirely through colony stimulating factor 1 receptor (CSF1R) inhibition are under investigation. CSF1R is a growth factor that TAMs depend on for differentiation and survival (156). Several small molecules have been developed against CSF1R, with observed tumor regression and extended survival upon treatment in several murine models of GBM (157, 158). When tested clinically in patients with recurrent GBM, PLX3397 (pexidartinib) alone did not increase PFS (23). Accordingly, combinations of PLX3397 with other immunotherapies, such as anti-PD1 and anti-CD40, are currently undergoing evaluation (159). MAbs that target CSF1R are also under investigation. Preclinical results with the novel anti-CSFR1 antibody RG7155 demonstrated death of CSF1R^+^ macrophages in patients with diffuse-type giant cell tumors. Additionally, there was a reduction in CD163^+^CSF1R^+^ macrophages in tumor tissue and increased CD8:CD4 ratio in response to treatment (24). Disappointingly in a separate phase 1b study of anti-CSF1R in patients with advanced solid tumors, no objective clinical response was observed (25). As with repolarization strategies, combining macrophage targeting therapies with ICI may improve efficacy.

#### 4.2.2 Targeting Tregs

Multiple approaches aimed at depleting Tregs have produced favorable outcomes in preclinical cancer models. CD25, the high affinity alpha chain of the IL-2 receptor, is constitutively expressed on the surface of Tregs and is often the target of choice for Treg manipulations. In one early study, Onizuka, et al. showed that depletion of Tregs in mice with the anti-CD25 antibody PC61 early after tumor implantation led to a significant regression of tumor size and an increase in survival (160). Importantly, this phenomenon was shown in several tumor models including leukemia, myeloma, and sarcoma (160) with other groups showing a similar effect in GBM (161). One major caveat of targeting Tregs through CD25 is that activated effector T cells transiently express CD25. Therefore, the true benefit of Treg depletion using anti-CD25 may be masked by concurrent depletion of anti-tumor effector T cells. Preclinical proof-of-concept studies in transgenic DEREG (DEpletion of REGulatory T cell) mice, whereby Tregs are depleted based on expression of the more faithful Treg marker Foxp3, have brought further insight. Early studies with DEREG mice showed that depleting Tregs *via* Foxp3 targeting resulted in partial regression of established B16 melanoma (162). This regression was accompanied by an increase in tumor infiltrating effector CD8 T cells. Subsequent studies using DEREG mice have confirmed the benefit of Treg depletion in other cancer models (163–166). However, depleting Tregs based on Foxp3 in humans is currently not possible due to its intracellular/intranuclear location as a transcription factor. Thus, clinical efforts mainly rely on manipulating the IL-2/CD25 axis to achieve Treg depletion.

Nevertheless, there are several strategies that rationally aim to deplete Tregs while sparing antitumor effector T cells. The common murine Treg-depleting antibody clone PC61 efficiently depletes CD25^hi^ Tregs but also blocks IL-2 signaling on remaining effector T cells thus blunting their effector activity. Solomon, et al. recently characterized a novel anti-CD25 antibody that depletes Tregs but does not block IL-2 signaling in effector T cells (αCD25^NIB^; NIB: Non-IL-2 Blocking) (17). This murine αCD25^NIB^ consists of a CD25 epitope-binding domain that is known to permit IL-2 signaling (clone 7D4) fused to the depleting murine IgG2a isotype backbone. αCD25^NIB^ promoted tumor rejection and prolonged survival in preclinical colorectal cancer models. Treg depletion with αCD25^NIB^ correlated with increased intratumoral effector T cell:Treg ratios along with increased granzyme B secretion by CD8 T cells. The anti-tumor effect was ablated when IL-2 was neutralized, signifying the dependency of IL-2 signaling for the anti-tumor activity of αCD25^NIB^. Importantly, these findings were translated into a human system, with similar Treg depleting, non-IL-2 blocking characteristics shown in non-human primates.

One major concern regarding systemic Treg depletion is the potential to induce autoimmunity, as Tregs are crucial for maintaining peripheral immune tolerance (167, 168). Thus, strategies designed specifically to deplete intratumoral Tregs only are emerging in an effort to assuage the risk of autoimmunity that can accompany systemic Treg depletion. One such approach involves injecting an immunotoxin-coupled anti-CD25 antibody (2E4-PE38) directly into the tumor (18). The authors elegantly showed that administering 2E4-PE38 directly into an established AB1 mesothelioma tumor led to tumor regression in both the injected tumor and a distal, non-injected tumor on the same mouse. These results indicated that local Treg depletion can potentially elicit concomitant tumor immunity. Importantly, the immunotoxin 2E4-PE38 has a relatively short half-life (<3 hours), allowing for effector cytotoxic T cells to repopulate the tumor quickly after CD25^+^ T cell depletion.

Another approach to deplete intratumoral Tregs targets the chemokine receptor CCR8. CCR8 is up-regulated on Tregs following activation in the presence of the CCR8 ligand CCL1. Signaling through CCR8 potentiates multiple Treg-associated immunosuppressive mechanisms including up-regulation of Foxp3, CD39, and IL-10 (169). Campbell, et al. provided evidence that CCR8 was more highly expressed by tumor-infiltrating Tregs than circulating Tregs from the same patient (16). Subsequently, they showed that treatment with a CCR8-targeted depleting antibody specifically depleted intratumoral Tregs and promoted a robust anti-tumor immune response. Importantly, CCR8-mediated depletion did not affect effector T cell populations.

Another recent approach targeting intratumoral Tregs utilized near-infrared photoimmunotherapy (NIR-PIT) to locally deplete Tregs (19). Briefly, NIR-PIT involves administering a monoclonal antibody or Fab conjugated to IR700 followed by exposure to near-infrared light (690 nm) (170). Kurebayashi, et al. used an anti-CD25-IR700 conjugate to selectively and rapidly kill Tregs after tumor exposure to NIR-light. In preclinical models of MC38 colon cancer and EO771 breast cancer, intratumoral Treg depletion was accompanied by increased intratumoral infiltration of CD8 T and NK cells and a decrease in tumor size in an IFN-γ dependent manner. However, tumors began to regrow concomitantly with Treg recovery. Thus, the kinetics of Treg depletion, effector T cell activation, and Treg repopulation must all be considered when depleting Tregs to enhance antitumor immunity.

Treg depletion has also been shown in some instances to negatively impact antitumor immunity (171). In a model of pancreatic cancer, depleting Tregs actually promoted carcinogenesis in part by increasing myeloid-derived suppressor cell infiltration and by modulating fibroblast architecture. This pro-tumor effect of Treg depletion is likely cancer-type dependent, but nonetheless, suggests other approaches to Treg manipulation may be worth pursuing. Depleting Tregs is not the only Treg-focused approach that can promote tumor clearance. An alternate strategy is to reprogram Tregs from suppressive T cells to effector T cells. Tregs are inherently unstable, meaning they can lose Foxp3 expression and gain effector function under certain conditions (e.g. inflammation) (172). This instability can be detrimental in the context of autoimmunity where strategies aim to stabilize Tregs (173) but can potentially be manipulated favorably in the context of cancer. Indeed, Amoozgar, *et al.* recently showed that treatment of mice bearing GBM with an agonist antibody targeting glucocorticoid-induced TNFR-related (GITR) protein redirected intratumoral Tregs to a CD4 effector lineage (20). GITR is constitutively expressed on Tregs and transiently on activated effector T cells (174). The function of GITR is cell-context-dependent but predominantly acts as a costimulatory signal (175). Previous studies using GITR agonism showed increased effector T cells activation and decreased Treg suppressive activity with resulting autoimmunity (176). However, the utilization of GITR agonism to repolarize Tregs to effector T cells is a novel and intriguing approach. The destabilized Tregs induced by anti-GITR agonism characteristically resembled Th1 cells, secreting IFN-γ and promoting tumor cytotoxicity. Anti-GITR significantly extended mouse survival even as a monotherapy, but it showed even further benefits when coupled with anti-PD1.

#### 4.2.3 Targeting Immunosuppressive Cytokines

Like conventional endogenous effector T cells, CAR T cells also prove susceptible to immunosuppressive mechanisms. TGF-β is a suppressive soluble factor present in high levels within various tumor types where it can prevent endogenous and CAR T cell activation. To combat this, Cadilha et al. generated a multicistronic epCAM-targeting CAR construct that encoded the chemokine receptor CCR8 and a dominant-negative TGF-β receptor (DNR) (177). CCR8 is uniquely upregulated on intratumoral Tregs (178) and, thus, forced expression of CCR8 enhanced CAR T cell trafficking to tumor while the DNR prevented suppression by TGF-β. These epCAM CCR8^+^ DNR CAR T cells were more efficacious at eliminating tumor and promoting survival in a preclinical model of pancreatic cancer than epCAM CAR T cells.

An interesting alternative approach to countering TGF-β (and suppressive cytokine signaling in general) involves converting the suppressive signal into a stimulatory one. Sukumaran, et al. designed and produced a prostate cancer antigen-targeting CAR that co-expressed TGF-β and IL-4 receptors in tandem with intracellular 4-1BB co-stimulatory domains and IL-7 receptor signaling domains, respectively (179). These “SmarT” cells were more potent and efficacious than normal CAR T cells in preclinical models, a benefit that was more pronounced in tumors designed to overexpress TGF-β and IL-4. Rational selection of intracellular signaling combinations to manipulate with this strategy should work to better rescue T cell activation.

#### 4.2.4 Targeting Hypoxia

In cytotoxic T cells, HIF-1α stabilization is induced in both hypoxia-dependent and -independent fashions. For instance, TCR activation itself can lead to increased HIF-1α signaling (66, 180). The HIF-1α signaling pathway appears to be critical to T cell effector function in the setting of hypoxia, as deletion of HIF-1α in T cells lead to decreased production of IFN-γ, granzyme B, and TNFα by CD8 T cells under hypoxic conditions, facilitating tumor growth (116) (**Figure 4**).

While HIF-1α stabilization within T cells may be beneficial to T cell function and the anti-tumor response, HIF-1α signaling within tumor cells can be problematic, fostering tumor survival within its hypoxic TME. In turn, inhibition of the HIF-1α signaling pathway in tumor cells has often been a therapeutic goal. Within tumor cells, HIF-1α pathway activation aids tumor survival and combats the anti-tumor immune response by upregulating tumor expression of PD-L1 and CD47. CD47 is a cell surface ligand that binds to signal regulatory protein α (SIRPα) on macrophages, serving as a “don’t eat me” signal (181, 182). HIF-1α additionally upregulates tumor autophagy, which is critical for tumor cell survival in the harsh metabolic conditions of the TME as it allows tumor cells to recycle damaged organelles and proteins into metabolic substrates for energy generation (181, 183–185). Ultimately, higher tumor expression of HIF-1α correlates with worse clinical outcome (186, 187).

HIF-1α therefore plays complex and potentially opposing roles in mediating tumor survival and the T cell antitumor response, and there are conflicting data as to whether systemic HIF-1α inhibition ultimately enhances or hinders cytotoxic T cell function and tumor growth. In a preclinical mouse study, selective deletion of HIF-1α in T cells led to increased tumor growth and decreased T cell tumor infiltration, survival, and effector function, measured by production of TNFα and IFNγ (116). On the contrary, in another study, reduced T cell HIF-1α expression was instead associated with improved CD8 memory cell formation and enhanced anti-tumor cytotoxicity of CD8 T cells (188). Clinical trials of HIF-1α inhibitors have thus far been disappointing, with many failing to produce objective responses (186, 189–191). Several recent and current studies are investigating the benefit of combination HIF-1α inhibition with the anti-VEGF drug bevacizumab (192, 193). The addition of bevacizumab appears to produce a modest clinical benefit, eliciting a 22% partial response in a phase I/II study of 22 RCC patients (192). In a phase II clinical trial of CRLX101, a nanoparticle inhibitor of HIF-1α, in recurrent ovarian cancer patients, the addition of bevacizumab improved ORR to 18% compared to 11% in CRLX101 monotherapy (193).

### 4.3 Combatting Exhaustion

#### 4.3.1 Novel ICI Strategies

To overcome limitations to current therapies, research is underway to identify novel ICI targets. Common targets include alternative inhibitory receptors that are phenotypically linked to T cell exhaustion, such as TIM3 and LAG3. Additionally, a recent study identified inhibitory receptor CD161 as an attractive novel target for ICI through single cell profiling of tumor-infiltrating T cells of patients with IDH-WT and IDH-mutant gliomas (7) (**Figure 1**).

TIM3 was initially described as a cell surface molecule on activated effector CD4 and cytotoxic CD8 T cells (194). TIM3 inhibits TCR signaling and can induce T cell death by signaling through tyrosine residues in its cytoplasmic tail upon binding one of its ligands (195). Major ligands for TIM3 include galectin-9 (Gal-9), and Ceacam-1, which are expressed on macrophages and some cancers, and CD4 T cells, respectively (196–198). More recently, TIM3 expression has been identified on innate immune cells (NK cells, DCs, and monocytes) (199–201), and importantly, terminally exhausted CD8 T cells (202). Identification of TIM3 in terminally exhausted cells was initially done in models of chronic infection, where TIM3 signified virus-specific T cells with the greatest defects in pro-inflammatory cytokine production. Blockade of TIM3 restored effector function of terminally exhausted T cells in LCMV, HCV, and HBV, where co-blockade with PD1 further enhanced T cell responses (203, 204). Additionally, in several cancers, including GBM, melanoma, and NSCLC, TIM3 has been used to identify infiltrating T cells with poor anti-tumor capabilities (50, 130). The blockade of TIM3 is complicated by its expression on several immune cells, where it functions uniquely. However, initial first-in-human phase 1/2 clinical trials in several solid tumors have shown promising results (NCT02817633, NCT02608268, NCT03099109). In NSCLC cancer patients that had previously progressed with anti-PD1 treatment alone, combination anti-TIM3 (TSR-022) and anti-PD1 (TSR-042) has shown antitumor activity, as well as safety and tolerability (8).

LAG3 is a member of the Ig superfamily of proteins, originally described in activated human NK and T cells (205). It has been shown to negatively regulate T cell proliferation, activation and effector function upon binding to one of its many ligands: major histocompatibility complex II (MHC II) on APCs, galactose-binding lectin (Gal-3) on a variety of tumor cells, and more recently, fibrinogen-like protein (FGL1), which is also present on solid tumors (9, 206, 207). Targeting LAG3 on T cells with antagonistic antibodies prevents downstream inhibitory signaling, but can also inhibit the suppressive activity of Tregs, which have constitutive expression of LAG3 (208, 209). Ongoing clinical trials with LAG3 as a monotherapy have thus far been disappointing, therefore combinatorial approaches with anti-PD1, anti-CTLA4, chemotherapies and chemoradiation are currently underway (NCT03459222, NCT04150965, NCT03978611). Of note, earlier this year, results from RELATIVITY-047 (NCT03470922), a phase 2/3 clinical trial testing the combination of anti-LAG3 (relatimab) and anti-PD1 (nivolumab) *vs*. nivolumab alone demonstrated extended progression free survival (PFS) (10.12 months *vs*. 4.63 months) in patients with advanced melanoma. Importantly, the combination was reasonably tolerated, with only 18.9% of patients experiencing grade 3-4 adverse side effects.

CD161 is encoded by killer cell lectin-like receptors subfamily B member 1 (*KLRB1*) and is expressed on human and murine T and NK cells (210). Importantly, murine CD161 was originally described as a family of seven NK receptors, known as the NKRP1 family; however, only a single human ortholog has been identified (211, 212). In 2005, C-type lectin domain family 2 member D (CLEC2D) was identified as a functional ligand for human CD161, similarly to murine NKRP1B and NKRP1D, suggesting a functional similarity to the human (213, 214). Investigators identified subsets of CD8 T cells that co-expressed common NK cell receptors, where high cytotoxicity signatures correlated with high NK cell receptor expression. Notably, CD161 was expressed on a larger fraction of infiltrating T cells than PD1 and was most highly expressed on clonally expanded CD8 T cells, effector CD4 T cells, but not Tregs.

Using tumor-specific T cells co-cultured with gliomaspheres (3-dimensional clusters of tumor cells derived from human patients), investigators showed that inactivation of *KLRB1* resulted in increased cytotoxic activity. Furthermore, in humanized mouse models, inactivation of *KLRB1* in tumor-infiltrating T cells led to enhanced anti-tumor killing, as evidenced by slower tumor progression, significantly increased survival time and increased infiltration of PD1^-^TIM3^-^ T cells compared to unaltered T cells. Importantly, using publicly available scRNAseq datasets, the *KLRB1* transcriptional program (genes that had significantly higher expression in *KLRB1^+^* T cells *vs. KLRB1^-^* T cells) was identified in tumor infiltrating T cells from multiple human cancers, including melanoma, NSCLC, hepatocellular carcinoma, and colorectal cancer (CRC). This highlights the CD161-CLEC2D axis for immunotherapeutic targeting more broadly. Researchers further suggested that monoclonal antibody targeting of CD161 could synergize with anti-PD1 treatment by targeting a larger group of infiltrating T cells because of the non-overlapping expression of the two targets. This study introduces the idea that inhibitory NK cell receptor expression on T cells represents a functionally relevant and targetable axis.

#### 4.3.2 Targeting Altered T Cell Metabolism

Therapeutic targets for countering tumor-imposed T cell metabolic and effector dysfunction include TNFR superfamily members 4-1BB and OX40. 4-1BB is an inducible costimulatory receptor that is normally highly expressed on activated CD4 and CD8 T cells, while being expressed at lower levels on NK cells, DCs, monocytes, and B cells. 4-1BB ligand, in turn, is expressed by macrophages, B cells and DCs (215–218).

4-1BB agonism has shown therapeutic promise due to its ability to prolong cytotoxic T cell activation and survival *in vivo* (1, 219). Specifically, 4-1BB improves the function and biogenesis of mitochondria in T cells, helping to improve the overall metabolic fitness of T cells (1, 181). Beyond its metabolic function, 4-1BB signaling also serves a proinflammatory role, activating NK-κB and ERK (1, 117). 4-1BB signaling has proven useful as a means of improving adoptive transfer therapies. The cytoplasmic signaling domain of 4-1BB was added to third generation CAR constructs and was demonstrated to improve both CAR T cell cytotoxicity and survival *in vivo* (220–223).

As a systemic monotherapy, 4-1BB agonism has been demonstrated to increase the anti-tumor response *in vivo* and *in vitro* in pre-clinical studies, though its application in clinical trials has been limited by significant dose-dependent hepatotoxicity. The most significant therapeutic potential for 4-1BB-directed therapy appears to be when it is used in conjunction with ICB. The improvements to metabolic function that occur as a result of activation of signaling pathways downstream of 4-1BB can synergize with the functional implications of blockade of PD1 or CTLA4 signaling networks, allowing activated effector T cells to better overcome the suppressive TME (217, 224). Our group and others have demonstrated the ability of 4-1BB agonism to license PD1 checkpoint blockade *in vivo* (217, 224–226). In our murine model of GBM, 4-1BB agonism + PD1 blockade produced 50% long term survival, whereas PD1 blockade alone offered no benefit, similar to failures that have been observed clinically with these tumors (226).

OX40 is a costimulatory receptor with similar functions to 4-1BB, ultimately promoting the effector function and survival of cytotoxic CD8 T cells (227). OX40 is expressed by activated CD4 and CD8 T cells. In CD8 T cells, OX40 enhances differentiation and proliferative expansion after priming (2). The metabolic effects of OX40 signaling on CD8 T cells are not well-elucidated thus far, though similarly to 4-1BB, OX40 has been shown to increase mitochondrial mass in CD8 T cells (228). Further, the OX40 and 4-1BB pathways appear to have considerable overlap, as combination 4-1BB and OX40 agonism therapy is additive, but not synergistic (217). As with 4-1BB, the improved effector T cell function resulting from OX40 signaling is synergistic with ICB therapy and has additionally been successfully utilized in second and third generation CAR constructs (217, 224, 229). OX40 also serves a role as a suppressor of Treg development and function, in part by blocking differentiation of CD4 T cells into Foxp3^+^ Tregs and by inhibiting regulatory activity of existing Tregs. Thus, OX40 agonism may produce multiple anti-tumor immune benefits (224, 227, 229).

#### 4.3.3 Reviving Exhausted CARs

CAR T cells are also subject to T cell exhaustion. This exhaustion can either be tumor-imposed or due to tonic signaling intrinsic to the CAR construct itself. Efforts to counteract tumor-imposed exhaustion include combining checkpoint blockade with CAR T cell therapy (230, 231), deleting coinhibitory molecules from CAR T cells (12–14), or generating CAR T cells that secrete checkpoint inhibitors (10, 11). Tonic signaling refers to ligand-independent CAR signaling that is caused by physical characteristics of the CAR construct itself (232). For example, high CAR expression on the surface of T cells can result in background activation signaling. This tonic signaling can induce T cell exhaustion that leads to suboptimal T cell activation in the presence of antigen.

Recently, Weber, et al. explored the effect of “resting” CAR T cells after viral transduction and prior to administration (15). The authors first engineered a GD2-targeting CAR with known problematic tonic signaling to contain an intracellular destabilization domain that required administration of a drug to induce surface CAR expression. CAR expression is thus restrained until tumor antigen is present, preventing CAR T cell exhaustion from tonic signaling. These CAR T cells were more efficacious *in vivo* in xenograft models. Interestingly, forcing “rest” after exhaustion had been imprinted appeared to reverse the exhaustion epigenetic profile. As an alternative approach, the Src kinase inhibitor dasatinib was used to transiently inhibit CAR T cell activation *in vitro* and *in vivo*, abrogating tonic signaling-induced exhaustion and promoting more potent antitumor immunity. Dasatinib allows for immediate applicability of this transient rest approach to a variety of CAR T cell therapies without the burden of redesigning new CAR constructs.

## 5 Discussion

The dramatic successes that can be observed with immunotherapies have made them a mainstay in the treatment of many cancers. Currently, however, such successes remain limited to a minority of cancer patients, highlighting the need for further research and innovation in this space. Emerging therapies are beginning to take a more complete view of the TME and are uncovering complementary strategies to simultaneously rescue and stimulate immune function and generate therapeutic synergism. For instance, T cell dysfunction exists in multiple forms within the TME, can have multiple sources, and can limit immunotherapeutic efficacy. We are beginning to understand more about the TME-driven origins of such dysfunction, and this understanding is increasingly and appropriately driving therapeutic design. T cell dysfunction can arise from lack of tumor-specificity, abnormal vasculature that limits trafficking, hypoxic/nutrient deprived environments, and immunosuppressive/pro-tumor cells (Tregs and macrophages). Targeting more rationally the mechanisms underlying these barriers to T cell function and combining such strategies for T cell reinvigoration with activating platforms such as ICI, have yielded striking preclinical and clinical results. These successes underscore the importance of a rational, multi-pronged immune-based approach to tumors and their microenvironment.

## Author Contributions

JW, EL, and DW conceived the manuscript. JW, EL, DW, and AH-M wrote the manuscript. JW, EL, and DW edited the manuscript. PF supervised all aspects of the work. All authors contributed to the article and approved the submitted version.

## Conflict of Interest

The authors declare that the research was conducted in the absence of any commercial or financial relationships that could be construed as a potential conflict of interest.

## Publisher’s Note

All claims expressed in this article are solely those of the authors and do not necessarily represent those of their affiliated organizations, or those of the publisher, the editors and the reviewers. Any product that may be evaluated in this article, or claim that may be made by its manufacturer, is not guaranteed or endorsed by the publisher.

## References

[1] Menk AV Scharping NE Rivadeneira DB Calderon MJ Watson MJ Dunstane D. 4-1BB Costimulation Induces T Cell Mitochondrial Function and Biogenesis Enabling Cancer Immunotherapeutic Responses. J Exp Med (2018) 215:1091–100. doi: 10.1084/jem.20171068 PMC5881463 29511066

[2] Redmond WL Ruby CE Weinberg AD. The Role of OX40-Mediated Co-Stimulation in T-Cell Activation and Survival. Crit Rev Immunol (2009) 29:187–201. doi: 10.1615/CritRevImmunol.v29.i3.10 19538134 PMC3180959

[3] Kashyap AS Schmittnaegel M Rigamonti N Pais-Ferreira D Mueller P Buchi M. Optimized Antiangiogenic Reprogramming of the Tumor Microenvironment Potentiates CD40 Immunotherapy. Proc Natl Acad Sci U S A (2020) 117:541–51. doi: 10.1073/pnas.1902145116 PMC6955310 31889004

[4] Stagg J Divisekera U McLaughlin N Sharkey J Pommey S Denoyer D. Anti-CD73 Antibody Therapy Inhibits Breast Tumor Growth and Metastasis. Proc Natl Acad Sci (2010) 107:1547–52. doi: 10.1073/pnas.0908801107 PMC2824381 20080644

[5] Murphy PS Wang J Bhagwat SP Munger JC Janssen WJ Wright TW. CD73 Regulates Anti-Inflammatory Signaling Between Apoptotic Cells and Endotoxin-Conditioned Tissue Macrophages. Cell Death Differ (2017) 24:559–70. doi: 10.1038/cdd.2016.159 PMC5344214 28060378

[6] Goswami S Walle T Cornish AE Basu S Anandhan S Fernandez I. Immune Profiling of Human Tumors Identifies CD73 as a Combinatorial Target in Glioblastoma. Nat Med (2020) 26:39–46. doi: 10.1038/s41591-019-0694-x 31873309 PMC7182038

[7] Mathewson ND Ashenberg O Tirosh I Gritsch S Perez EM Marx S. Inhibitory CD161 Receptor Identified in Glioma-Infiltrating T Cells by Single-Cell Analysis. Cell (2021) 184:1281–98.e26. doi: 10.1016/j.cell.2021.01.022 33592174 PMC7935772

[8] Acharya N Sabatos-Peyton C Anderson AC. Tim-3 Finds its Place in the Cancer Immunotherapy Landscape. J Immunother Cancer (2020) 8(1):e000911. doi: 10.1136/jitc-2020-000911 32601081 PMC7326247

[9] Wang J Sanmamed MF Datar I Su TT Ji L Sun J. Fibrinogen-Like Protein 1 Is a Major Immune Inhibitory Ligand of LAG-3. Cell (2019) 176:334–47.e12. doi: 10.1016/j.cell.2018.11.010 30580966 PMC6365968

[10] Suarez ER Chang de K Sun J Sui J Freeman GJ Signoretti S. Chimeric Antigen Receptor T Cells Secreting Anti-PD-L1 Antibodies More Effectively Regress Renal Cell Carcinoma in a Humanized Mouse Model. Oncotarget (2016) 7:34341–55. doi: 10.18632/oncotarget.9114 PMC5085160 27145284

[11] Li S Siriwon N Zhang X Yang S Jin T He F. Enhanced Cancer Immunotherapy by Chimeric Antigen Receptor-Modified T Cells Engineered to Secrete Checkpoint Inhibitors. Clin Cancer Res (2017) 23:6982–92. doi: 10.1158/1078-0432.CCR-17-0867 28912137

[12] Rupp LJ Schumann K Roybal KT Gate RE Ye CJ Lim WA. CRISPR/Cas9-Mediated PD-1 Disruption Enhances Anti-Tumor Efficacy of Human Chimeric Antigen Receptor T Cells. Sci Rep (2017) 7:737. doi: 10.1038/s41598-017-00462-8 28389661 PMC5428439

[13] Hu W Zi Z Jin Y Li G Shao K Cai Q. CRISPR/Cas9-Mediated PD-1 Disruption Enhances Human Mesothelin-Targeted CAR T Cell Effector Functions. Cancer Immunol Immunother (2019) 68:365–77. doi: 10.1007/s00262-018-2281-2 PMC11028344 30523370

[14] Choi BD Yu X Castano AP Darr H Henderson DB Bouffard AA. CRISPR-Cas9 Disruption of PD-1 Enhances Activity of Universal EGFRvIII CAR T Cells in a Preclinical Model of Human Glioblastoma. J Immunother Cancer (2019) 7:304. doi: 10.1186/s40425-019-0806-7 31727131 PMC6857271

[15] Weber EW Parker KR Sotillo E Lynn RC Anbunathan H Lattin J. Transient Rest Restores Functionality in Exhausted CAR-T Cells Through Epigenetic Remodeling. Science (2021) 372(6537):eaba1786. doi: 10.1126/science.aba1786 33795428 PMC8049103

[16] Campbell JR McDonald BR Mesko PB Siemers NO Singh PB Selby M. Fc-Optimized Anti-CCR8 Antibody Depletes Regulatory T Cells in Human Tumor Models. Cancer Res (2021) 81:2983–94. doi: 10.1158/0008-5472.CAN-20-3585 33757978

[17] Solomon I Amann M Goubier A Vargas FA Zervas D Qing C. CD25-Treg-Depleting Antibodies Preserving IL-2 Signaling on Effector T Cells Enhance Effector Activation and Antitumor Immunity. Nat Cancer (2020) 1:1153–66. doi: 10.1038/s43018-020-00133-0 PMC7116816 33644766

[18] Onda M Kobayashi K Pastan I. Depletion of Regulatory T Cells in Tumors With an Anti-CD25 Immunotoxin Induces CD8 T Cell-Mediated Systemic Antitumor Immunity. Proc Natl Acad Sci U S A (2019) 116:4575–82. doi: 10.1073/pnas.1820388116 PMC6410866 30760587

[19] Kurebayashi Y Olkowski CP Lane KC Vasalatiy OV Xu BC Okada R. Rapid Depletion of Intratumoral Regulatory T Cells Induces Synchronized CD8 T- and NK-Cell Activation and IFNgamma-Dependent Tumor Vessel Regression. Cancer Res (2021) 81:3092–104. doi: 10.1158/0008-5472.CAN-20-2673 PMC8178213 33574087

[20] Amoozgar Z Kloepper J Ren J Tay RE Kazer SW Kiner E. Targeting Treg Cells With GITR Activation Alleviates Resistance to Immunotherapy in Murine Glioblastomas. Nat Commun (2021) 12:2582. doi: 10.1038/s41467-021-22885-8 33976133 PMC8113440

[21] Scholz A Harter PN Cremer S Yalcin BH Gurnik S Yamaji M. Endothelial Cell-Derived Angiopoietin-2 is a Therapeutic Target in Treatment-Naive and Bevacizumab-Resistant Glioblastoma. EMBO Mol Med (2016) 8:39–57. doi: 10.15252/emmm.201505505 26666269 PMC4718155

[22] Flores-Toro JA Luo D Gopinath A Sarkisian MR Campbell JJ Charo IF. CCR2 Inhibition Reduces Tumor Myeloid Cells and Unmasks a Checkpoint Inhibitor Effect to Slow Progression of Resistant Murine Gliomas. Proc Natl Acad Sci U S A (2020) 117:1129–38. doi: 10.1073/pnas.1910856117 PMC6969504 31879345

[23] Butowski N Colman H De Groot JF Omuro AM Nayak L Wen PY. Orally Administered Colony Stimulating Factor 1 Receptor Inhibitor PLX3397 in Recurrent Glioblastoma: An Ivy Foundation Early Phase Clinical Trials Consortium Phase II Study. Neuro Oncol (2016) 18:557–64. doi: 10.1093/neuonc/nov245 PMC4799682 26449250

[24] Ries CH Cannarile MA Hoves S Benz J Wartha K Runza V. Targeting Tumor-Associated Macrophages With Anti-CSF-1R Antibody Reveals a Strategy for Cancer Therapy. Cancer Cell (2014) 25:846–59. doi: 10.1016/j.ccr.2014.05.016 24898549

[25] Machiels JP Gomez-Roca C Michot JM Zamarin D Mitchell T Catala G. Phase Ib Study of Anti-CSF-1R Antibody Emactuzumab in Combination With CD40 Agonist Selicrelumab in Advanced Solid Tumor Patients. J Immunother Cancer (2020) 8:e001153. doi: 10.1136/jitc-2020-001153 33097612 PMC7590375

[26] Grasso CS Tsoi J Onyshchenko M Abril-Rodriguez G Ross-Macdonald P Wind-Rotolo M. Conserved Interferon-γ Signaling Drives Clinical Response to Immune Checkpoint Blockade Therapy in Melanoma. Cancer Cell (2020) 38:500–15.e3. doi: 10.1158/1538-7445.AM2020-3166 32916126 PMC7872287

[27] Zhang S Kohli K Black RG Yao L Spadinger SM He Q. Systemic Interferon-γ Increases MHC Class I Expression and T-Cell Infiltration in Cold Tumors: Results of a Phase 0 Clinical Trial. Cancer Immunol Res (2019) 7:1237–43. doi: 10.1158/2326-6066.CIR-18-0940 PMC6677581 31171504

[28] Choi BD Yu X Castano AP Bouffard AA Schmidts A Larson RC. CAR-T Cells Secreting BiTEs Circumvent Antigen Escape Without Detectable Toxicity. Nat Biotechnol (2019) 37:1049–58. doi: 10.1038/s41587-019-0192-1 31332324

[29] Choe JH Watchmaker PB Simic MS Gilbert RD Li AW Krasnow NA. SynNotch-CAR T Cells Overcome Challenges of Specificity, Heterogeneity, and Persistence in Treating Glioblastoma. Sci Transl Med (2021) 13(591):eabe7378. doi: 10.1126/scitranslmed.abe7378 33910979 PMC8362330

[30] Hyrenius-Wittsten A Su Y Park M Garcia JM Alavi J Perry N. SynNotch CAR Circuits Enhance Solid Tumor Recognition and Promote Persistent Antitumor Activity in Mouse Models. Sci Transl Med (2021) 13(591):eabd8836. doi: 10.1126/scitranslmed.abd8836 33910981 PMC8594452

[31] Keir ME Butte MJ Freeman GJ Sharpe AH. PD-1 and its Ligands in Tolerance and Immunity. Annu Rev Immunol (2008) 26:677–704. doi: 10.1146/annurev.immunol.26.021607.090331 18173375 PMC10637733

[32] YERVOY (ipilimumab) Injection approval letter. in: U.S.F.a.D. (2011).

[33] O'Day SJ Hamid O Urba WJ. Targeting Cytotoxic T-Lymphocyte Antigen-4 (CTLA-4): A Novel Strategy for the Treatment of Melanoma and Other Malignancies. Cancer (2007) 110:2614–27. doi: 10.1002/cncr.23086 18000991

[34] Chemnitz JM Parry RV Nichols KE June CH Riley JL. SHP-1 and SHP-2 Associate With Immunoreceptor Tyrosine-Based Switch Motif of Programmed Death 1 Upon Primary Human T Cell Stimulation, But Only Receptor Ligation Prevents T Cell Activation. J Immunol (2004) 173:945–54. doi: 10.4049/jimmunol.173.2.945 15240681

[35] Hamid O Robert C Daud A Hodi FS Hwu WJ Kefford R. Five-Year Survival Outcomes for Patients With Advanced Melanoma Treated With Pembrolizumab in KEYNOTE-001. Ann Oncol (2019) 30:582–8. doi: 10.1093/annonc/mdz011 PMC6503622 30715153

[36] Robert C Ribas A Hamid O Daud A Wolchok JD Joshua AM. Durable Complete Response After Discontinuation of Pembrolizumab in Patients With Metastatic Melanoma. J Clin Oncol (2018) 36:1668–74. doi: 10.1200/JCO.2017.75.6270 29283791

[37] Gong J Chehrazi-Raffle A Reddi S Salgia R. Development of PD-1 and PD-L1 Inhibitors as a Form of Cancer Immunotherapy: A Comprehensive Review of Registration Trials and Future Considerations. J Immunother Cancer (2018) 6:8. doi: 10.1186/s40425-018-0316-z 29357948 PMC5778665

[38] Sandru A Voinea S Panaitescu E Blidaru A. Survival Rates of Patients With Metastatic Malignant Melanoma. J Med Life (2014) 7:572–6. PMC4316142 25713625

[39] Larkin J Chiarion-Sileni V Gonzalez R Grob J-J Rutkowski P Lao CD. Five-Year Survival With Combined Nivolumab and Ipilimumab in Advanced Melanoma. N Engl J Med (2019) 381:1535–46. doi: 10.1056/NEJMoa1910836 31562797

[40] Pitt JM Vétizou M Daillère R Roberti MaríaP Yamazaki T Routy B. Resistance Mechanisms to Immune-Checkpoint Blockade in Cancer: Tumor-Intrinsic and -Extrinsic Factors. Immunity (2016) 44:1255–69. doi: 10.1016/j.immuni.2016.06.001 27332730

[41] Haslam A Prasad V. Estimation of the Percentage of US Patients With Cancer Who Are Eligible for and Respond to Checkpoint Inhibitor Immunotherapy Drugs. JAMA Network Open (2019) 2:e192535–e192535. doi: 10.1001/jamanetworkopen.2019.2535 31050774 PMC6503493

[42] Haslam A Gill J Prasad V. Estimation of the Percentage of US Patients With Cancer Who Are Eligible for Immune Checkpoint Inhibitor Drugs. JAMA Network Open (2020) 3:e200423–e200423. doi: 10.1001/jamanetworkopen.2020.0423 32150268 PMC7063495

[43] Shergold AL Millar R Nibbs RJB. Understanding and Overcoming the Resistance of Cancer to PD-1/PD-L1 Blockade. Pharmacol Res (2019) 145:104258. doi: 10.1016/j.phrs.2019.104258 31063806

[44] Eshhar Z Waks T Gross G Schindler DG. Specific Activation and Targeting of Cytotoxic Lymphocytes Through Chimeric Single Chains Consisting of Antibody-Binding Domains and the Gamma or Zeta Subunits of the Immunoglobulin and T-Cell Receptors. Proc Natl Acad Sci U S A (1993) 90:720–4. doi: 10.1073/pnas.90.2.720 PMC45737 8421711

[45] Holstein SA Lunning MA. CAR T-Cell Therapy in Hematologic Malignancies: A Voyage in Progress. Clin Pharmacol Ther (2020) 107:112–22. doi: 10.1002/cpt.1674 31622496

[46] Gentles AJ Newman AM Liu CL Bratman SV Feng W Kim D. The Prognostic Landscape of Genes and Infiltrating Immune Cells Across Human Cancers. Nat Med (2015) 21:938–45. doi: 10.1038/nm.3909 PMC4852857 26193342

[47] Quail DF Joyce JA. The Microenvironmental Landscape of Brain Tumors. Cancer Cell (2017) 31:326–41. doi: 10.1016/j.ccell.2017.02.009 PMC5424263 28292436

[48] Tsujikawa T Kumar S Borkar RN Azimi V Thibault G Chang YH. Quantitative Multiplex Immunohistochemistry Reveals Myeloid-Inflamed Tumor-Immune Complexity Associated With Poor Prognosis. Cell Rep (2017) 19:203–17. doi: 10.1016/j.celrep.2017.03.037 PMC5564306 28380359

[49] Beatty GL Winograd R Evans RA Long KB Luque SL Lee JW. Exclusion of T Cells From Pancreatic Carcinomas in Mice Is Regulated by Ly6C(low) F4/80(+) Extratumoral Macrophages. Gastroenterology (2015) 149:201–10. doi: 10.1053/j.gastro.2015.04.010 PMC4478138 25888329

[50] Woroniecka K Chongsathidkiet P Rhodin K Kemeny H Dechant C Farber SH. T-Cell Exhaustion Signatures Vary With Tumor Type and Are Severe in Glioblastoma. Clin Cancer Res (2018) 24:4175–86. doi: 10.1158/1078-0432.CCR-17-1846 PMC6081269 29437767

[51] Thorsson V Gibbs DL Brown SD Wolf D Bortone DS Ou Yang TH. The Immune Landscape of Cancer. Immunity (2018) 48:812–30.e14. doi: 10.1016/j.immuni.2018.03.023 29628290 PMC5982584

[52] Aird WC. Phenotypic Heterogeneity of the Endothelium: I. Structure, Function, and Mechanisms. Circ Res (2007) 100:158–73. doi: 10.1161/01.RES.0000255691.76142.4a 17272818

[53] Owens T Bechmann I Engelhardt B. Perivascular Spaces and the Two Steps to Neuroinflammation. J Neuropathol Exp Neurol (2008) 67:1113–21. doi: 10.1097/NEN.0b013e31818f9ca8 19018243

[54] Tietz S Engelhardt B. Brain Barriers: Crosstalk Between Complex Tight Junctions and Adherens Junctions. J Cell Biol (2015) 209:493–506. doi: 10.1083/jcb.201412147 26008742 PMC4442813

[55] De Palma M Biziato D Petrova TV. Microenvironmental Regulation of Tumour Angiogenesis. Nat Rev Cancer (2017) 17:457–74. doi: 10.1038/nrc.2017.51 28706266

[56] DeNardo DG Ruffell B. Macrophages as Regulators of Tumour Immunity and Immunotherapy. Nat Rev Immunol (2019) 19:369–82. doi: 10.1038/s41577-019-0127-6 PMC7339861 30718830

[57] Peranzoni E Lemoine J Vimeux L Feuillet V Barrin S Kantari-Mimoun C. Macrophages Impede CD8 T Cells From Reaching Tumor Cells and Limit the Efficacy of Anti-PD-1 Treatment. Proc Natl Acad Sci U S A (2018) 115:E4041–50. doi: 10.1073/pnas.1720948115 PMC5924916 29632196

[58] Quaranta V Rainer C Nielsen SR Raymant ML Ahmed MS Engle DD. Macrophage-Derived Granulin Drives Resistance to Immune Checkpoint Inhibition in Metastatic Pancreatic Cancer. Cancer Res (2018) 78:4253–69. doi: 10.1158/0008-5472.CAN-17-3876 PMC6076440 29789416

[59] Mariathasan S Turley SJ Nickles D Castiglioni A Yuen K Wang Y. Powles, TGFbeta Attenuates Tumour Response to PD-L1 Blockade by Contributing to Exclusion of T Cells. Nature (2018) 554:544–8. doi: 10.1038/nature25501 PMC6028240 29443960

[60] Tauriello DVF Palomo-Ponce S Stork D Berenguer-Llergo A Badia-Ramentol J Iglesias M. TGFbeta Drives Immune Evasion in Genetically Reconstituted Colon Cancer Metastasis. Nature (2018) 554:538–43. doi: 10.1038/nature25492 29443964

[61] Kelly A Gunaltay S McEntee CP Shuttleworth EE Smedley C Houston SA. Human Monocytes and Macrophages Regulate Immune Tolerance *via* Integrin Alphavbeta8-Mediated TGFbeta Activation. J Exp Med (2018) 215:2725–36. doi: 10.1084/jem.20171491 PMC6219736 30355614

[62] Naito Y Saito K Shiiba K Ohuchi A Saigenji K Nagura H. CD8+ T Cells Infiltrated Within Cancer Cell Nests as a Prognostic Factor in Human Colorectal Cancer. Cancer Res (1998) 58:3491–4. 9721846

[63] Zhang L Conejo-Garcia JR Katsaros D Gimotty PA Massobrio M Regnani G. Intratumoral T Cells, Recurrence, and Survival in Epithelial Ovarian Cancer. N Engl J Med (2003) 348:203–13. doi: 10.1056/NEJMoa020177 12529460

[64] Cornel AM Mimpen IL Nierkens S. MHC Class I Downregulation in Cancer: Underlying Mechanisms and Potential Targets for Cancer Immunotherapy. Cancers (Basel) (2020) 12(7):1760. doi: 10.3390/cancers12071760 PMC7409324 32630675

[65] Zaretsky JM Garcia-Diaz A Shin DS Escuin-Ordinas H Hugo W Hu-Lieskovan S. Mutations Associated With Acquired Resistance to PD-1 Blockade in Melanoma. N Engl J Med (2016) 375:819–29. doi: 10.1056/NEJMoa1604958 PMC5007206 27433843

[66] Chisolm DA Weinmann AS. TCR-Signaling Events in Cellular Metabolism and Specialization. Front Immunol (2015) 6. doi: 10.3389/fimmu.2015.00292 PMC4459085 26106392

[67] Bubeník J. Tumour MHC Class I Downregulation and Immunotherapy (Review). Oncol Rep (2003) 10:2005–8. doi: 10.3892/or.10.6.2005 14534734

[68] Peng M Mo Y Wang Y Wu P Zhang Y Xiong F. Neoantigen Vaccine: An Emerging Tumor Immunotherapy. Mol Cancer (2019) 18:128. doi: 10.1186/s12943-019-1055-6 31443694 PMC6708248

[69] Zhao P Li L Jiang X Li Q. Mismatch Repair Deficiency/Microsatellite Instability-High as a Predictor for Anti-PD-1/PD-L1 Immunotherapy Efficacy. J Hematol Oncol (2019) 12:54. doi: 10.1186/s13045-019-0738-1 31151482 PMC6544911

[70] Schumacher TN Schreiber RD. Neoantigens in Cancer Immunotherapy. Science (2015) 348:69–74. doi: 10.1126/science.aaa4971 25838375

[71] van Rooij N van Buuren MM Philips D Velds A Toebes M Heemskerk B. Tumor Exome Analysis Reveals Neoantigen-Specific T-Cell Reactivity in an Ipilimumab-Responsive Melanoma. J Clin Oncol (2013) 31:e439–42. doi: 10.1200/JCO.2012.47.7521 PMC3836220 24043743

[72] Sharma P Allison JP. The Future of Immune Checkpoint Therapy. Science (2015) 348:56–61. doi: 10.1126/science.aaa8172 25838373

[73] Rich BS Honeyman JN Darcy DG Smith PT Williams AR Lim IIP. Endogenous Antibodies for Tumor Detection. Sci Rep (2014) 4:5088. doi: 10.1038/srep05088 24875800 PMC4038850

[74] Merck. Keytruda: Highlights of Prescribing Information (2014). U.S. Food and Drug Administration (Accessed July 2021). website Revised March 2021.

[75] Kato T Kiyotani K Tomiyama E Koh Y Matsushita M Hayashi Y. Peripheral T Cell Receptor Repertoire Features Predict Durable Responses to Anti-PD-1 Inhibitor Monotherapy in Advanced Renal Cell Carcinoma. Oncoimmunology (2021) 10:1862948. doi: 10.1080/2162402X.2020.1862948 33537170 PMC7833759

[76] Poran A Scherer J Bushway ME Besada R Balogh KN Wanamaker A. Combined TCR Repertoire Profiles and Blood Cell Phenotypes Predict Melanoma Patient Response to Personalized Neoantigen Therapy Plus Anti-PD-1. Cell Rep Med (2020) 1(8):100141. doi: 10.1016/j.xcrm.2020.100141 33294862 PMC7691446

[77] Han J Duan J Bai H Wang Y Wan R Wang X. TCR Repertoire Diversity of Peripheral PD-1+CD8+ T Cells Predicts Clinical Outcomes After Immunotherapy in Patients With Non–Small Cell Lung Cancer. Cancer Immunol Res (2020) 8:146–54. doi: 10.1158/2326-6066.CIR-19-0398 31719056

[78] Patel AP Tirosh I Trombetta JJ Shalek AK Gillespie SM Wakimoto H. Single-Cell RNA-Seq Highlights Intratumoral Heterogeneity in Primary Glioblastoma. Science (2014) 344:1396–401. doi: 10.1126/science.1254257 PMC4123637 24925914

[79] Sampson JH Heimberger AB Archer GE Aldape KD Friedman AH Friedman HS. Immunologic Escape After Prolonged Progression-Free Survival With Epidermal Growth Factor Receptor Variant III Peptide Vaccination in Patients With Newly Diagnosed Glioblastoma. J Clin Oncol (2010) 28:4722–9. doi: 10.1200/JCO.2010.28.6963 PMC3020702 20921459

[80] Maude SL Laetsch TW Buechner J Rives S Boyer M Bittencourt H. Tisagenlecleucel in Children and Young Adults With B-Cell Lymphoblastic Leukemia. N Engl J Med (2018) 378:439–48. doi: 10.1056/NEJMoa1709866 PMC5996391 29385370

[81] Ruella M Maus MV. Catch Me If You can: Leukemia Escape After CD19-Directed T Cell Immunotherapies. Comput Struct Biotechnol J (2016) 14:357–62. doi: 10.1016/j.csbj.2016.09.003 PMC5061074 27761200

[82] Carter SL Eklund AC Kohane IS Harris LN Szallasi Z. A Signature of Chromosomal Instability Inferred From Gene Expression Profiles Predicts Clinical Outcome in Multiple Human Cancers. Nat Genet (2006) 38:1043–8. doi: 10.1038/ng1861 16921376

[83] Burrell RA McGranahan N Bartek J Swanton C. The Causes and Consequences of Genetic Heterogeneity in Cancer Evolution. Nature (2013) 501:338–45. doi: 10.1038/nature12625 24048066

[84] Raynaud F Mina M Tavernari D Ciriello G. Pan-Cancer Inference of Intra-Tumor Heterogeneity Reveals Associations With Different Forms of Genomic Instability. PLoS Genet (2018) 14:e1007669. doi: 10.1371/journal.pgen.1007669 30212491 PMC6155543

[85] Sottoriva A Spiteri I Piccirillo SG Touloumis A Collins VP Marioni JC. Intratumor Heterogeneity in Human Glioblastoma Reflects Cancer Evolutionary Dynamics. Proc Natl Acad Sci U S A (2013) 110:4009–14. doi: 10.1073/pnas.1219747110 PMC3593922 23412337

[86] Gerlinger M Horswell S Larkin J Rowan AJ Salm MP Varela I. Genomic Architecture and Evolution of Clear Cell Renal Cell Carcinomas Defined by Multiregion Sequencing. Nat Genet (2014) 46:225–33. doi: 10.1038/ng.2891 PMC4636053 24487277

[87] Bowman RL Klemm F Akkari L Pyonteck SM Sevenich L Quail DF. Macrophage Ontogeny Underlies Differences in Tumor-Specific Education in Brain Malignancies. Cell Rep (2016) 17:2445–59. doi: 10.1016/j.celrep.2016.10.052 PMC5450644 27840052

[88] Chen Z Feng X Herting CJ Garcia VA Nie K Pong WW. Cellular and Molecular Identity of Tumor-Associated Macrophages in Glioblastoma. Cancer Res (2017) 77:2266–78. doi: 10.1158/0008-5472.CAN-16-2310 PMC5741820 28235764

[89] Zhu Y Herndon JM Sojka DK Kim KW Knolhoff BL Zuo C. Tissue-Resident Macrophages in Pancreatic Ductal Adenocarcinoma Originate From Embryonic Hematopoiesis and Promote Tumor Progression. Immunity (2017) 47:597. doi: 10.1016/j.immuni.2017.08.018 28930665 PMC5664180

[90] Opitz CA Litzenburger UM Sahm F Ott M Tritschler I Trump S. An Endogenous Tumour-Promoting Ligand of the Human Aryl Hydrocarbon Receptor. Nature (2011) 478:197–203. doi: 10.1038/nature10491 21976023

[91] Gajewski TF Schreiber H Fu YX. Innate and Adaptive Immune Cells in the Tumor Microenvironment. Nat Immunol (2013) 14:1014–22. doi: 10.1038/ni.2703 PMC4118725 24048123

[92] Noy R Pollard JW. Tumor-Associated Macrophages: From Mechanisms to Therapy. Immunity (2014) 41:49–61. doi: 10.1016/j.immuni.2014.06.010 25035953 PMC4137410

[93] De Palma M Venneri MA Galli R Sergi Sergi L Politi LS Sampaolesi M. Tie2 Identifies a Hematopoietic Lineage of Proangiogenic Monocytes Required for Tumor Vessel Formation and a Mesenchymal Population of Pericyte Progenitors. Cancer Cell (2005) 8:211–26. doi: 10.1016/j.ccr.2005.08.002 16169466

[94] Mazzieri R Pucci F Moi D Zonari E Ranghetti A Berti A. Targeting the ANG2/TIE2 Axis Inhibits Tumor Growth and Metastasis by Impairing Angiogenesis and Disabling Rebounds of Proangiogenic Myeloid Cells. Cancer Cell (2011) 19:512–26. doi: 10.1016/j.ccr.2011.02.005 21481792

[95] Wildin RS Ramsdell F Peake J Faravelli F Casanova JL Buist N. X-Linked Neonatal Diabetes Mellitus, Enteropathy and Endocrinopathy Syndrome is the Human Equivalent of Mouse Scurfy. Nat Genet (2001) 27:18–20. doi: 10.1038/83707 11137992

[96] Bennett CL Christie J Ramsdell F Brunkow ME Ferguson PJ Whitesell L. The Immune Dysregulation, Polyendocrinopathy, Enteropathy, X-Linked Syndrome (IPEX) Is Caused by Mutations of FOXP3. Nat Genet (2001) 27:20–1. doi: 10.1038/83713 11137993

[97] Huan J Culbertson N Spencer L Bartholomew R Burrows GG Chou YK. Decreased FOXP3 Levels in Multiple Sclerosis Patients. J Neurosci Res (2005) 81:45–52. doi: 10.1002/jnr.20522 15952173

[98] Balandina A Lecart S Dartevelle P Saoudi A Berrih-Aknin S. Functional Defect of Regulatory CD4(+)CD25+ T Cells in the Thymus of Patients With Autoimmune Myasthenia Gravis. Blood (2005) 105:735–41. doi: 10.1182/blood-2003-11-3900 PMC1847365 15454488

[99] Lindley S Dayan CM Bishop A Roep BO Peakman M Tree TI. Defective Suppressor Function in CD4(+)CD25(+) T-Cells From Patients With Type 1 Diabetes. Diabetes (2005) 54:92–9. doi: 10.2337/diabetes.54.1.92 15616015

[100] Fecci PE Mitchell DA Whitesides JF Xie W Friedman AH Archer GE. Increased Regulatory T-Cell Fraction Amidst a Diminished CD4 Compartment Explains Cellular Immune Defects in Patients With Malignant Glioma. Cancer Res (2006) 66:3294–302. doi: 10.1158/0008-5472.CAN-05-3773 16540683

[101] Chaudhary B Abd Al Samid M al-Ramadi BK Elkord E. Phenotypic Alterations, Clinical Impact and Therapeutic Potential of Regulatory T Cells in Cancer. Expert Opin Biol Ther (2014) 14:931–45. doi: 10.1517/14712598.2014.900539 24661020

[102] Ichihara F Kono K Takahashi A Kawaida H Sugai H Fujii H. Increased Populations of Regulatory T Cells in Peripheral Blood and Tumor-Infiltrating Lymphocytes in Patients With Gastric and Esophageal Cancers. Clin Cancer Res (2003) 9:4404–8. 14555512

[103] Liyanage UK Moore TT Joo HG Tanaka Y Herrmann V Doherty G. Prevalence of Regulatory T Cells Is Increased in Peripheral Blood and Tumor Microenvironment of Patients With Pancreas or Breast Adenocarcinoma. J Immunol (2002) 169:2756–61. doi: 10.4049/jimmunol.169.5.2756 12193750

[104] Mourmouras V Fimiani M Rubegni P Epistolato MC Malagnino V Cardone C. Evaluation of Tumour-Infiltrating CD4+CD25+FOXP3+ Regulatory T Cells in Human Cutaneous Benign and Atypical Naevi, Melanomas and Melanoma Metastases. Br J Dermatol (2007) 157:531–9. doi: 10.1111/j.1365-2133.2007.08057.x 17596146

[105] Plitas G Konopacki C Wu K Bos PD Morrow M Putintseva EV. Regulatory T Cells Exhibit Distinct Features in Human Breast Cancer. Immunity (2016) 45:1122–34. doi: 10.1016/j.immuni.2016.10.032 PMC5134901 27851913

[106] Oderup C Cederbom L Makowska A Cilio CM Ivars F. Cytotoxic T Lymphocyte Antigen-4-Dependent Down-Modulation of Costimulatory Molecules on Dendritic Cells in CD4+ CD25+ Regulatory T-Cell-Mediated Suppression. Immunology (2006) 118:240–9. doi: 10.1111/j.1365-2567.2006.02362.x PMC1782280 16771859

[107] Fahlen L Read S Gorelik L Hurst SD Coffman RL Flavell RA. T Cells That Cannot Respond to TGF-Beta Escape Control by CD4(+)CD25(+) Regulatory T Cells. J Exp Med (2005) 201:737–46. doi: 10.1084/jem.20040685 PMC2212836 15753207

[108] Asseman C Mauze S Leach MW Coffman RL Powrie F. An Essential Role for Interleukin 10 in the Function of Regulatory T Cells That Inhibit Intestinal Inflammation. J Exp Med (1999) 190:995–1004. doi: 10.1084/jem.190.7.995 10510089 PMC2195650

[109] Pandiyan P Zheng L Ishihara S Reed J Lenardo MJ. CD4+CD25+Foxp3+ Regulatory T Cells Induce Cytokine Deprivation-Mediated Apoptosis of Effector CD4+ T Cells. Nat Immunol (2007) 8:1353–62. doi: 10.1038/ni1536 17982458

[110] Vignali DA Collison LW Workman CJ. How Regulatory T Cells Work. Nat Rev Immunol (2008) 8:523–32. doi: 10.1038/nri2343 PMC2665249 18566595

[111] Scharping NE Rivadeneira DB Menk AV Vignali PDA Ford BR Rittenhouse NL. Mitochondrial Stress Induced by Continuous Stimulation Under Hypoxia Rapidly Drives T Cell Exhaustion. Nat Immunol (2021) 22:205–15. doi: 10.1038/s41590-020-00834-9 PMC7971090 33398183

[112] Liu YN Yang JF Huang DJ Ni HH Zhang CX Zhang L. Hypoxia Induces Mitochondrial Defect That Promotes T Cell Exhaustion in Tumor Microenvironment Through MYC-Regulated Pathways. Front Immunol (2020) 11:1906. doi: 10.3389/fimmu.2020.01906 32973789 PMC7472844

[113] Semenza GL. Hypoxia-Inducible Factor 1: Regulator of Mitochondrial Metabolism and Mediator of Ischemic Preconditioning. Biochim Biophys Acta (2011) 1813:1263–8. doi: 10.1016/j.bbamcr.2010.08.006 PMC3010308 20732359

[114] Roman J Rangasamy T Guo J Sugunan S Meednu N Packirisamy G. T-Cell Activation Under Hypoxic Conditions Enhances IFN-Gamma Secretion. Am J Respir Cell Mol Biol (2010) 42:123–8. doi: 10.1165/rcmb.2008-0139OC PMC2809218 19372249

[115] Semenza GL Roth PH Fang HM Wang GL. Transcriptional Regulation of Genes Encoding Glycolytic Enzymes by Hypoxia-Inducible Factor 1. J Biol Chem (1994) 269:23757–63. doi: 10.1016/S0021-9258(17)31580-6 8089148

[116] Palazon A Tyrakis PA Macias D Veliça P Rundqvist H Fitzpatrick S. An HIF-1α/VEGF-A Axis in Cytotoxic T Cells Regulates Tumor Progression. Cancer Cell (2017) 32:669–83.e5. doi: 10.1016/j.ccell.2017.10.003 29136509 PMC5691891

[117] Teijeira A Garasa S Etxeberria I Gato-Cañas M Melero I Delgoffe GM. Metabolic Consequences of T-Cell Costimulation in Anticancer Immunity. Cancer Immunol Res (2019) 7:1564–9. doi: 10.1158/2326-6066.CIR-19-0115 31575551

[118] Koyama S Akbay EA Li YY Herter-Sprie GS Buczkowski KA Richards WG. Adaptive Resistance to Therapeutic PD-1 Blockade Is Associated With Upregulation of Alternative Immune Checkpoints. Nat Commun (2016) 7:10501. doi: 10.1038/ncomms10501 26883990 PMC4757784

[119] Angelosanto JM Blackburn SD Crawford A Wherry EJ. Progressive Loss of Memory T Cell Potential and Commitment to Exhaustion During Chronic Viral Infection. J Virol (2012) 86:8161–70. doi: 10.1128/JVI.00889-12 PMC3421680 22623779

[120] Pauken KE Sammons MA Odorizzi PM Manne S Godec J Khan O. Epigenetic Stability of Exhausted T Cells Limits Durability of Reinvigoration by PD-1 Blockade. Science (2016) 354:1160–5. doi: 10.1126/science.aaf2807 PMC5484795 27789795

[121] Sen DR Kaminski J Barnitz RA Kurachi M Gerdemann U Yates KB. The Epigenetic Landscape of T Cell Exhaustion. Science (2016) 354:1165–9. doi: 10.1126/science.aae0491 PMC5497589 27789799

[122] McLane LM Abdel-Hakeem MS Wherry EJ. CD8 T Cell Exhaustion During Chronic Viral Infection and Cancer. Annu Rev Immunol (2019) 37:457–95. doi: 10.1146/annurev-immunol-041015-055318 30676822

[123] Miggelbrink AM Jackson JD Lorrey SJ Srinivasan ES Waibl Polania J Wilkinson DS. CD4 T-Cell Exhaustion: Does it Exist and What are its Roles in Cancer? Clin Cancer Res (2021) 27(21):5742–52. doi: 10.1158/1078-0432.CCR-21-0206 PMC8563372 34127507

[124] Im SJ Hashimoto M Gerner MY Lee J Kissick HT Burger MC. Defining CD8+ T Cells That Provide the Proliferative Burst After PD-1 Therapy. Nature (2016) 537:417–21. doi: 10.1038/nature19330 PMC5297183 27501248

[125] Wu T Ji Y Moseman EA Xu HC Manglani M Kirby M. The TCF1-Bcl6 Axis Counteracts Type I Interferon to Repress Exhaustion and Maintain T Cell Stemness. Sci Immunol (2016) 1(6):eaai8593. doi: 10.1126/sciimmunol.aai8593 28018990 PMC5179228

[126] Utzschneider DT Charmoy M Chennupati V Pousse L Ferreira DP Calderon-Copete S. T Cell Factor 1-Expressing Memory-Like CD8(+) T Cells Sustain the Immune Response to Chronic Viral Infections. Immunity (2016) 45:415–27. doi: 10.1016/j.immuni.2016.07.021 27533016

[127] Leong YA Chen Y Ong HS Wu D Man K Deleage C. CXCR5(+) Follicular Cytotoxic T Cells Control Viral Infection in B Cell Follicles. Nat Immunol (2016) 17:1187–96. doi: 10.1038/ni.3543 27487330

[128] He R Hou S Liu C Zhang A Bai Q Han M. Follicular CXCR5- Expressing CD8(+) T Cells Curtail Chronic Viral Infection. Nature (2016) 537:412–28. doi: 10.1038/nature19317 27501245

[129] Chen Z Ji Z Ngiow SF Manne S Cai Z Huang AC. TCF-1-Centered Transcriptional Network Drives an Effector *Versus* Exhausted CD8 T Cell-Fate Decision. Immunity (2019) 51:840–55.e5. doi: 10.1016/j.immuni.2019.09.013 31606264 PMC6943829

[130] Miller BC Sen DR Al Abosy R Bi K Virkud YV LaFleur MW. Subsets of Exhausted CD8(+) T Cells Differentially Mediate Tumor Control and Respond to Checkpoint Blockade. Nat Immunol (2019) 20:326–36. doi: 10.1038/s41590-019-0312-6 PMC6673650 30778252

[131] Blackburn SD Shin H Freeman GJ Wherry EJ. Selective Expansion of a Subset of Exhausted CD8 T Cells by alphaPD-L1 Blockade. Proc Natl Acad Sci U S A (2008) 105:15016–21. doi: 10.1073/pnas.0801497105 PMC2567485 18809920

[132] Guo Y Xie YQ Gao M Zhao Y Franco F Wenes M. Metabolic Reprogramming of Terminally Exhausted CD8(+) T Cells by IL-10 Enhances Anti-Tumor Immunity. Nat Immunol (2021) 22:746–56. doi: 10.1038/s41590-021-00940-2 PMC7610876 34031618

[133] Kim KJ Li B Winer J Armanini M Gillett N Phillips HS. Inhibition of Vascular Endothelial Growth Factor-Induced Angiogenesis Suppresses Tumour Growth *In Vivo* . Nature (1993) 362:841–4. doi: 10.1038/362841a0 7683111

[134] Socinski MA Jotte RM Cappuzzo F Orlandi F Stroyakovskiy D Nogami N. Atezolizumab for First-Line Treatment of Metastatic Nonsquamous NSCLC. N Engl J Med (2018) 378:2288–301. doi: 10.1056/NEJMoa1716948 29863955

[135] Song E Mao T Dong H Boisserand LSB Antila S Bosenberg M. VEGF-C-Driven Lymphatic Drainage Enables Immunosurveillance of Brain Tumours. Nature (2020) 577:689–94. doi: 10.1038/s41586-019-1912-x PMC7100608 31942068

[136] Skobe M Hawighorst T Jackson DG Prevo R Janes L Velasco P. Induction of Tumor Lymphangiogenesis by VEGF-C Promotes Breast Cancer Metastasis. Nat Med (2001) 7:192–8. doi: 10.1038/84643 11175850

[137] Mathur SP Mathur RS Gray EA Lane D Underwood PG Kohler M. Serum Vascular Endothelial Growth Factor C (VEGF-C) as a Specific Biomarker for Advanced Cervical Cancer: Relationship to Insulin-Like Growth Factor II (IGF-II), IGF Binding Protein 3 (IGF-BP3) and VEGF-A [Corrected]. Gynecol Oncol (2005) 98:467–83. doi: 10.1016/j.ygyno.2005.05.003 15982726

[138] Curtsinger JM Lins DC Mescher MF. Signal 3 Determines Tolerance *Versus* Full Activation of Naive CD8 T Cells: Dissociating Proliferation and Development of Effector Function. J Exp Med (2003) 197:1141–51. doi: 10.1084/jem.20021910 PMC2193970 12732656

[139] Leonard JP Sherman ML Fisher GL Buchanan LJ Larsen G Atkins MB. Effects of Single-Dose Interleukin-12 Exposure on Interleukin-12-Associated Toxicity and Interferon-Gamma Production. Blood (1997) 90:2541–8. 9326219

[140] Liu Y Di S Shi B Zhang H Wang Y Wu X. Armored Inducible Expression of IL-12 Enhances Antitumor Activity of Glypican-3-Targeted Chimeric Antigen Receptor-Engineered T Cells in Hepatocellular Carcinoma. J Immunol (2019) 203:198–207. doi: 10.4049/jimmunol.1800033 31142602

[141] Vonderheide RH. The Immune Revolution: A Case for Priming, Not Checkpoint. Cancer Cell (2018) 33:563–9. doi: 10.1016/j.ccell.2018.03.008 PMC5898647 29634944

[142] Nowak AK Robinson BW Lake RA. Synergy Between Chemotherapy and Immunotherapy in the Treatment of Established Murine Solid Tumors. Cancer Res (2003) 63:4490–6. 12907622

[143] O'Hara MH O'Reilly EM Varadhachary G Wolff RA Wainberg ZA Ko AH. CD40 Agonistic Monoclonal Antibody APX005M (Sotigalimab) and Chemotherapy, With or Without Nivolumab, for the Treatment of Metastatic Pancreatic Adenocarcinoma: An Open-Label, Multicentre, Phase 1b Study. Lancet Oncol (2021) 22:118–31. doi: 10.1016/S1470-2045(20)30532-5 33387490

[144] Bajor DL Mick R Riese MJ Huang AC Sullivan B Richman LP. Long-Term Outcomes of a Phase I Study of Agonist CD40 Antibody and CTLA-4 Blockade in Patients With Metastatic Melanoma. Oncoimmunology (2018) 7:e1468956. doi: 10.1080/2162402X.2018.1468956 30288340 PMC6169575

[145] Jin D Fan J Wang L Thompson LF Liu A Daniel BJ. CD73 on Tumor Cells Impairs Antitumor T-Cell Responses: A Novel Mechanism of Tumor-Induced Immune Suppression. Cancer Res (2010) 70:2245–55. doi: 10.1158/0008-5472.CAN-09-3109 PMC2883609 20179192

[146] Moesta AK Li X-Y Smyth MJ. Targeting CD39 in Cancer. Nat Rev Immunol (2020) 20:739–55. doi: 10.1038/s41577-020-0376-4 32728220

[147] Ohta A Gorelik E Prasad SJ Ronchese F Lukashev D Wong MKK. A2A Adenosine Receptor Protects Tumors From Antitumor T Cells. Proc Natl Acad Sci (2006) 103:13132–7. doi: 10.1073/pnas.0605251103 PMC1559765 16916931

[148] Lin H Wei S Hurt EM Green MD Zhao L Vatan L. Host Expression of PD-L1 Determines Efficacy of PD-L1 Pathway Blockade–Mediated Tumor Regression. J Clin Invest (2018) 128:805–15. doi: 10.1172/JCI96113 PMC5785251 29337305

[149] Häusler SF Montalbán del Barrio I Strohschein J Chandran PA Engel JB Hönig A. Ectonucleotidases CD39 and CD73 on OvCA Cells are Potent Adenosine-Generating Enzymes Responsible for Adenosine Receptor 2A-Dependent Suppression of T Cell Function and NK Cell Cytotoxicity. Cancer Immunol Immunother (2011) 60:1405–18. doi: 10.1007/s00262-011-1040-4 PMC11028787 21638125

[150] Li D-K Wang W. Characteristics and Clinical Trial Results of Agonistic Anti−CD40 Antibodies in the Treatment of Malignancies (Review). Oncol Lett (2020) 20:176. doi: 10.3892/ol.2020.12037 32934743 PMC7471753

[151] Harvey JB Phan LH Villarreal OE Bowser JL. CD73's Potential as an Immunotherapy Target in Gastrointestinal Cancers. Front Immunol (2020) 11:508. doi: 10.3389/fimmu.2020.00508 32351498 PMC7174602

[152] Li L Liu YD Zhan YT Zhu YH Li Y Xie D. High Levels of CCL2 or CCL4 in the Tumor Microenvironment Predict Unfavorable Survival in Lung Adenocarcinoma. Thorac Cancer (2018) 9:775–84. doi: 10.1111/1759-7714.12643 PMC6026602 29722145

[153] Ueno T Toi M Saji H Muta M Bando H Kuroi K. Significance of Macrophage Chemoattractant Protein-1 in Macrophage Recruitment, Angiogenesis, and Survival in Human Breast Cancer. Clin Cancer Res (2000) 6:3282–9. 10955814

[154] Li X Yao W Yuan Y Chen P Li B Li J. Targeting of Tumour-Infiltrating Macrophages *via* CCL2/CCR2 Signalling as a Therapeutic Strategy Against Hepatocellular Carcinoma. Gut (2017) 66:157–67. doi: 10.1136/gutjnl-2015-310514 26452628

[155] Tu MM Abdel-Hafiz HA Jones RT Jean A Hoff KJ Duex JE. Inhibition of the CCL2 Receptor, CCR2, Enhances Tumor Response to Immune Checkpoint Therapy. Commun Biol (2020) 3:720. doi: 10.1038/s42003-020-01441-y 33247183 PMC7699641

[156] Patel S Player MR. Colony-Stimulating Factor-1 Receptor Inhibitors for the Treatment of Cancer and Inflammatory Disease. Curr Top Med Chem (2009) 9:599–610. doi: 10.2174/156802609789007327 19689368

[157] Pyonteck SM Akkari L Schuhmacher AJ Bowman RL Sevenich L Quail DF. CSF-1R Inhibition Alters Macrophage Polarization and Blocks Glioma Progression. Nat Med (2013) 19:1264–72. doi: 10.1038/nm.3337 PMC3840724 24056773

[158] Przystal JM Becker H Canjuga D Tsiami F Anderle N Keller AL. Targeting CSF1R Alone or in Combination With PD1 in Experimental Glioma. Cancers (Basel) (2021) 13(10):2400. doi: 10.3390/cancers13102400 34063518 PMC8156558

[159] Li DK Wang W. Characteristics and Clinical Trial Results of Agonistic Anti-CD40 Antibodies in the Treatment of Malignancies. Oncol Lett (2020) 20:176. doi: 10.3892/ol.2020.12037 32934743 PMC7471753

[160] Onizuka S Tawara I Shimizu J Sakaguchi S Fujita T Nakayama E. Tumor Rejection by *In Vivo* Administration of Anti-CD25 (Interleukin-2 Receptor Alpha) Monoclonal Antibody. Cancer Res (1999) 59:3128–33. 10397255

[161] Fecci PE Sweeney AE Grossi PM Nair SK Learn CA Mitchell DA. Systemic Anti-CD25 Monoclonal Antibody Administration Safely Enhances Immunity in Murine Glioma Without Eliminating Regulatory T Cells. Clin Cancer Res (2006) 12:4294–305. doi: 10.1158/1078-0432.CCR-06-0053 16857805

[162] Klages K Mayer CT Lahl K Loddenkemper C Teng MW Ngiow SF. Selective Depletion of Foxp3+ Regulatory T Cells Improves Effective Therapeutic Vaccination Against Established Melanoma. Cancer Res (2010) 70:7788–99. doi: 10.1158/0008-5472.CAN-10-1736 20924102

[163] Akhmetzyanova I Zelinskyy G Schimmer S Brandau S Altenhoff P Sparwasser T. Tumor-Specific CD4+ T Cells Develop Cytotoxic Activity and Eliminate Virus-Induced Tumor Cells in the Absence of Regulatory T Cells. Cancer Immunol Immunother (2013) 62:257–71. doi: 10.1007/s00262-012-1329-y PMC3569596 22890822

[164] Fisher SA Aston WJ Chee J Khong A Cleaver AL Solin JN. Transient Treg Depletion Enhances Therapeutic Anti-Cancer Vaccination. Immun Inflammation Dis (2017) 5:16–28. doi: 10.1002/iid3.136 PMC5322183 28250921

[165] Ji L Qian W Gui L Ji Z Yin P Lin GN. Blockade of Beta-Catenin-Induced CCL28 Suppresses Gastric Cancer Progression *via* Inhibition of Treg Cell Infiltration. Cancer Res (2020) 80:2004–16. doi: 10.1158/0008-5472.CAN-19-3074 32156780

[166] Mattarollo SR Steegh K Li M Duret H Foong Ngiow S Smyth MJ. Transient Foxp3(+) Regulatory T-Cell Depletion Enhances Therapeutic Anticancer Vaccination Targeting the Immune-Stimulatory Properties of NKT Cells. Immunol Cell Biol (2013) 91:105–14. doi: 10.1038/icb.2012.58 23090488

[167] Ellis JS Wan X Braley-Mullen H. Transient Depletion of CD4+ CD25+ Regulatory T Cells Results in Multiple Autoimmune Diseases in Wild-Type and B-Cell-Deficient NOD Mice. Immunology (2013) 139:179–86. doi: 10.1111/imm.12065 PMC3647184 23293979

[168] Tanaka A Sakaguchi S. Regulatory T Cells in Cancer Immunotherapy. Cell Res (2017) 27:109–18. doi: 10.1038/cr.2016.151 PMC5223231 27995907

[169] Barsheshet Y Wildbaum G Levy E Vitenshtein A Akinseye C Griggs J. CCR8(+)FOXp3(+) Treg Cells as Master Drivers of Immune Regulation. Proc Natl Acad Sci U S A (2017) 114:6086–91. doi: 10.1073/pnas.1621280114 PMC5468670 28533380

[170] Kobayashi H Furusawa A Rosenberg A Choyke PL. Near-Infrared Photoimmunotherapy of Cancer: A New Approach That Kills Cancer Cells and Enhances Anti-Cancer Host Immunity. Int Immunol (2021) 33:7–15. doi: 10.1093/intimm/dxaa037 32496557 PMC7771006

[171] Zhang Y Lazarus J Steele NG Yan W Lee HJ Nwosu ZC. Regulatory T-Cell Depletion Alters the Tumor Microenvironment and Accelerates Pancreatic Carcinogenesis. Cancer Discov (2020) 10:422–39. doi: 10.1158/2159-8290.CD-19-0958 PMC7224338 31911451

[172] Overacre AE Vignali DA. T(reg) Stability: To be or Not to be. Curr Opin Immunol (2016) 39:39–43. doi: 10.1016/j.coi.2015.12.009 26774863 PMC4801724

[173] Wilkinson DS Ghosh D Nickle RA Moorman CD Mannie MD. Partial CD25 Antagonism Enables Dominance of Antigen-Inducible CD25(high) FOXP3(+) Regulatory T Cells As a Basis for a Regulatory T Cell-Based Adoptive Immunotherapy. Front Immunol (2017) 8:1782. doi: 10.3389/fimmu.2017.01782 29312311 PMC5735073

[174] McHugh RS Whitters MJ Piccirillo CA Young DA Shevach EM Collins M. CD4(+)CD25(+) Immunoregulatory T Cells: Gene Expression Analysis Reveals a Functional Role for the Glucocorticoid-Induced TNF Receptor. Immunity (2002) 16:311–23. doi: 10.1016/S1074-7613(02)00280-7 11869690

[175] Ronchetti S Zollo O Bruscoli S Agostini M Bianchini R Nocentini G. GITR, a Member of the TNF Receptor Superfamily, is Costimulatory to Mouse T Lymphocyte Subpopulations. Eur J Immunol (2004) 34:613–22. doi: 10.1002/eji.200324804 14991590

[176] Shimizu J Yamazaki S Takahashi T Ishida Y Sakaguchi S. Stimulation of CD25(+)CD4(+) Regulatory T Cells Through GITR Breaks Immunological Self-Tolerance. Nat Immunol (2002) 3:135–42. doi: 10.1038/ni759 11812990

[177] Cadilha BL Benmebarek MR Dorman K Oner A Lorenzini T Obeck H. Combined Tumor-Directed Recruitment and Protection From Immune Suppression Enable CAR T Cell Efficacy in Solid Tumors. Sci Adv (2021) 7(24):eabi5781. doi: 10.1126/sciadv.abi5781 34108220 PMC8189699

[178] De Simone M Arrigoni A Rossetti G Gruarin P Ranzani V Politano C. Transcriptional Landscape of Human Tissue Lymphocytes Unveils Uniqueness of Tumor-Infiltrating T Regulatory Cells. Immunity (2016) 45:1135–47. doi: 10.1016/j.immuni.2016.10.021 PMC5119953 27851914

[179] Sukumaran S Watanabe N Bajgain P Raja K Mohammed S Fisher WE. Enhancing the Potency and Specificity of Engineered T Cells for Cancer Treatment. Cancer Discovery (2018) 8:972–87. doi: 10.1158/2159-8290.CD-17-1298 PMC6428579 29880586

[180] Tao JH Barbi J Pan F. Hypoxia-Inducible Factors in T Lymphocyte Differentiation and Function. A Review in the Theme: Cellular Responses to Hypoxia. Am J Physiol Cell Physiol (2015) 309:C580–9. doi: 10.1152/ajpcell.00204.2015 PMC4628938 26354751

[181] Noman MZ Hasmim M Lequeux A Xiao M Duhem C Chouaib S. Improving Cancer Immunotherapy by Targeting the Hypoxic Tumor Microenvironment: New Opportunities and Challenges. Cells (2019) 8(9):1083. doi: 10.3390/cells8091083 PMC6770817 31540045

[182] Barsoum IB Smallwood CA Siemens DR Graham CH. A Mechanism of Hypoxia-Mediated Escape From Adaptive Immunity in Cancer Cells. Cancer Res (2014) 74:665–74. doi: 10.1158/0008-5472.CAN-13-0992 24336068

[183] Wu H Huang S Chen Z Liu W Zhou X Zhang D. Hypoxia-Induced Autophagy Contributes to the Invasion of Salivary Adenoid Cystic Carcinoma Through the HIF-1α/BNIP3 Signaling Pathway. Mol Med Rep (2015) 12:6467–74. doi: 10.3892/mmr.2015.4255 PMC4626194 26323347

[184] Wang P Long M Zhang S Cheng Z Zhao X He F. Hypoxia Inducible Factor-1α Regulates Autophagy *via* the P27-E2F1 Signaling Pathway. Mol Med Rep (2017) 16:2107–12. doi: 10.3892/mmr.2017.6794 PMC5562089 28627618

[185] Mathew R Karantza-Wadsworth V White E. Role of Autophagy in Cancer. Nat Rev Cancer (2007) 7:961–7. doi: 10.1038/nrc2254 PMC2866167 17972889

[186] Fallah J Rini BI. HIF Inhibitors: Status of Current Clinical Development. Curr Oncol Rep (2019) 21:6. doi: 10.1007/s11912-019-0752-z 30671662

[187] Zhang L Ye SB Li ZL Ma G Chen SP He J. Increased HIF-1alpha Expression in Tumor Cells and Lymphocytes of Tumor Microenvironments Predicts Unfavorable Survival in Esophageal Squamous Cell Carcinoma Patients. Int J Clin Exp Pathol (2014) 7:3887–97. PMC4129000 25120765

[188] Sukumar M Liu J Ji Y Subramanian M Crompton JG Yu Z. Inhibiting Glycolytic Metabolism Enhances CD8+ T Cell Memory and Antitumor Function. J Clin Invest (2013) 123:4479–88. doi: 10.1172/JCI69589 PMC3784544 24091329

[189] Garrett CR Bekaii-Saab TS Ryan T Fisher GA Clive S Kavan P. Randomized Phase 2 Study of Pegylated SN-38 (EZN-2208) or Irinotecan Plus Cetuximab in Patients With Advanced Colorectal Cancer. Cancer (2013) 119:4223–30. doi: 10.1002/cncr.28358 24105075

[190] Chao J Lin J Frankel P Clark AJ Wiley DT Garmey E. Pilot Trial of CRLX101 in Patients With Advanced, Chemotherapy-Refractory Gastroesophageal Cancer. J Gastrointest Oncol (2017) 8:962–9. doi: 10.21037/jgo.2017.08.10 PMC5750185 29299355

[191] Matei D Schilder J Sutton G Perkins S Breen T Quon C. Activity of 2 Methoxyestradiol (Panzem® NCD) in Advanced, Platinum-Resistant Ovarian Cancer and Primary Peritoneal Carcinomatosis: A Hoosier Oncology Group Trial. Gynecologic Oncol (2009) 115:90–6. doi: 10.1016/j.ygyno.2009.05.042 19577796

[192] Keefe SM Hoffman-Censits J Cohen RB Mamtani R Heitjan D Eliasof S. Efficacy of the Nanoparticle–Drug Conjugate CRLX101 in Combination With Bevacizumab in Metastatic Renal Cell Carcinoma: Results of an Investigator-Initiated Phase I–IIa Clinical Trial. Ann Oncol (2016) 27:1579–85. doi: 10.1093/annonc/mdw188 PMC4959924 27457310

[193] Krasner CN Campos SM Young CL Chadda KR Lee H Birrer MJ. Sequential Phase II Clinical Trials Evaluating CRLX101 as Monotherapy and in Combination With Bevacizumab in Recurrent Ovarian Cancer. Gynecol Oncol (2021) 162(3):661–6. doi: 10.1016/j.ygyno.2021.07.002 34243976

[194] Monney L Sabatos CA Gaglia JL Ryu A Waldner H Chernova T. Th1-Specific Cell Surface Protein Tim-3 Regulates Macrophage Activation and Severity of an Autoimmune Disease. Nature (2002) 415:536–41. doi: 10.1038/415536a 11823861

[195] Zhu C Anderson AC Schubart A Xiong H Imitola J Khoury SJ. The Tim-3 Ligand Galectin-9 Negatively Regulates T Helper Type 1 Immunity. Nat Immunol (2005) 6:1245–52. doi: 10.1038/ni1271 16286920

[196] Seki M Sakata KM Oomizu S Arikawa T Sakata A Ueno M. Beneficial Effect of Galectin 9 on Rheumatoid Arthritis by Induction of Apoptosis of Synovial Fibroblasts. Arthritis Rheum (2007) 56:3968–76. doi: 10.1002/art.23076 18050192

[197] Wiener Z Kohalmi B Pocza P Jeager J Tolgyesi G Toth S. TIM-3 is Expressed in Melanoma Cells and is Upregulated in TGF-Beta Stimulated Mast Cells. J Invest Dermatol (2007) 127:906–14. doi: 10.1038/sj.jid.5700616 17096021

[198] Huang YH Zhu C Kondo Y Anderson AC Gandhi A Russell A. CEACAM1 Regulates TIM-3-Mediated Tolerance and Exhaustion. Nature (2015) 517:386–90. doi: 10.1038/nature13848 PMC4297519 25363763

[199] da Silva IP Gallois A Jimenez-Baranda S Khan S Anderson AC Kuchroo VK. Reversal of NK-Cell Exhaustion in Advanced Melanoma by Tim-3 Blockade. Cancer Immunol Res (2014) 2:410–22. doi: 10.1158/2326-6066.CIR-13-0171 PMC4046278 24795354

[200] Anderson AC Anderson DE Bregoli L Hastings WD Kassam N Lei C. Promotion of Tissue Inflammation by the Immune Receptor Tim-3 Expressed on Innate Immune Cells. Science (2007) 318:1141–3. doi: 10.1126/science.1148536 18006747

[201] Dixon KO Tabaka M Schramm MA Xiao S Tang R Dionne D. TIM-3 Restrains Anti-Tumour Immunity by Regulating Inflammasome Activation. Nature (2021) 595:101–6. doi: 10.1038/s41586-021-03626-9 PMC8627694 34108686

[202] Sakuishi K Apetoh L Sullivan JM Blazar BR Kuchroo VK Anderson AC. Targeting Tim-3 and PD-1 Pathways to Reverse T Cell Exhaustion and Restore Anti-Tumor Immunity. J Exp Med (2010) 207:2187–94. doi: 10.1084/jem.20100643 PMC2947065 20819927

[203] Jin HT Anderson AC Tan WG West EE Ha SJ Araki K. Cooperation of Tim-3 and PD-1 in CD8 T-Cell Exhaustion During Chronic Viral Infection. Proc Natl Acad Sci U S A (2010) 107:14733–8. doi: 10.1073/pnas.1009731107 PMC2930455 20679213

[204] McMahan RH Golden-Mason L Nishimura MI McMahon BJ Kemper M Allen TM. Tim-3 Expression on PD-1+ HCV-Specific Human CTLs Is Associated With Viral Persistence, and its Blockade Restores Hepatocyte-Directed *In Vitro* Cytotoxicity. J Clin Invest (2010) 120:4546–57. doi: 10.1172/JCI43127 PMC2994339 21084749

[205] Triebel F Jitsukawa S Baixeras E Roman-Roman S Genevee C Viegas-Pequignot E. LAG-3, a Novel Lymphocyte Activation Gene Closely Related to CD4. J Exp Med (1990) 171:1393–405. doi: 10.1084/jem.171.5.1393 PMC2187904 1692078

[206] Huard B Tournier M Hercend T Triebel F Faure F. Lymphocyte-Activation Gene 3/Major Histocompatibility Complex Class II Interaction Modulates the Antigenic Response of CD4+ T Lymphocytes. Eur J Immunol (1994) 24:3216–21. doi: 10.1002/eji.1830241246 7805750

[207] Workman CJ Dugger KJ Vignali DA. Cutting Edge: Molecular Analysis of the Negative Regulatory Function of Lymphocyte Activation Gene-3. J Immunol (2002) 169:5392–5. doi: 10.4049/jimmunol.169.10.5392 12421911

[208] Huang CT Workman CJ Flies D Pan X Marson AL Zhou G. Role of LAG-3 in Regulatory T Cells. Immunity (2004) 21:503–13. doi: 10.1016/j.immuni.2004.08.010 15485628

[209] Gagliani N Magnani CF Huber S Gianolini ME Pala M Licona-Limon P. Coexpression of CD49b and LAG-3 Identifies Human and Mouse T Regulatory Type 1 Cells. Nat Med (2013) 19:739–46. doi: 10.1038/nm.3179 23624599

[210] Lanier LL Chang C Phillips JH. Human NKR-P1A. A Disulfide-Linked Homodimer of the C-Type Lectin Superfamily Expressed by a Subset of NK and T Lymphocytes. J Immunol (1994) 153:2417–28. 8077657

[211] Aust JG Gays F Mickiewicz KM Buchanan E Brooks CG. The Expression and Function of the NKRP1 Receptor Family in C57BL/6 Mice. J Immunol (2009) 183:106–16. doi: 10.4049/jimmunol.0804281 19535641

[212] Glimcher L Shen FW Cantor H. Identification of a Cell-Surface Antigen Selectively Expressed on the Natural Killer Cell. J Exp Med (1977) 145:1–9. doi: 10.1084/jem.145.1.1 187714 PMC2180593

[213] Aldemir H Prod'homme V Dumaurier MJ Retiere C Poupon G Cazareth J. Cutting Edge: Lectin-Like Transcript 1 Is a Ligand for the CD161 Receptor. J Immunol (2005) 175:7791–5. doi: 10.4049/jimmunol.175.12.7791 16339512

[214] Rosen DB Bettadapura J Alsharifi M Mathew PA Warren HS Lanier LL. Cutting Edge: Lectin-Like Transcript-1 Is a Ligand for the Inhibitory Human NKR-P1A Receptor. J Immunol (2005) 175:7796–9. doi: 10.4049/jimmunol.175.12.7796 16339513

[215] Moran AE Kovacsovics-Bankowski M Weinberg AD. The TNFRs OX40, 4-1BB, and CD40 as Targets for Cancer Immunotherapy. Curr Opin Immunol (2013) 25:230–7. doi: 10.1016/j.coi.2013.01.004 PMC3815601 23414607

[216] Vinay DS Kwon BS. 4-1bb (CD137), an Inducible Costimulatory Receptor, as a Specific Target for Cancer Therapy. BMB Rep (2014) 47:122–9. doi: 10.5483/BMBRep.2014.47.3.283 PMC4163883 24499671

[217] Lee S-J Myers L Muralimohan G Dai J Qiao Y Li Z. 4-1BB and OX40 Dual Costimulation Synergistically Stimulate Primary Specific CD8 T Cells for Robust Effector Function. J Immunol (2004) 173:3002–12. doi: 10.4049/jimmunol.173.5.3002 15322159

[218] Melero I Johnston JV Shufford WW Mittler RS Chen L. NK1.1 Cells Express 4-1BB (CDw137) Costimulatory Molecule and Are Required for Tumor Immunity Elicited by Anti-4-1BB Monoclonal Antibodies. Cell Immunol (1998) 190:167–72. doi: 10.1006/cimm.1998.1396 9878117

[219] Vinay DS Kwon BS. 4-1BB Signaling Beyond T Cells. Cell Mol Immunol (2011) 8:281–4. doi: 10.1038/cmi.2010.82 PMC4002439 21217771

[220] Kawalekar OU O’Connor RS Fraietta JA Guo L McGettigan SE Posey AD. Distinct Signaling of Coreceptors Regulates Specific Metabolism Pathways and Impacts Memory Development in CAR T Cells. Immunity (2016) 44:380–90. doi: 10.1016/j.immuni.2016.01.021 PMC13498396 26885860

[221] Dotti G Gottschalk S Savoldo B Brenner MK. Design and Development of Therapies Using Chimeric Antigen Receptor-Expressing T Cells. Immunol Rev (2014) 257:107–26. doi: 10.1111/imr.12131 PMC3874724 24329793

[222] Imai C Mihara K Andreansky M Nicholson IC Pui CH Geiger TL. Chimeric Receptors With 4-1BB Signaling Capacity Provoke Potent Cytotoxicity Against Acute Lymphoblastic Leukemia. Leukemia (2004) 18:676–84. doi: 10.1038/sj.leu.2403302 14961035

[223] Sadelain M Brentjens R Rivière I. The Basic Principles of Chimeric Antigen Receptor Design. Cancer Discov (2013) 3:388–98. doi: 10.1158/2159-8290.CD-12-0548 PMC3667586 23550147

[224] Deng J Zhao S Zhang X Jia K Wang H Zhou C. OX40 (CD134) and OX40 Ligand, Important Immune Checkpoints in Cancer. Onco Targets Ther (2019) 12:7347–53. doi: 10.2147/OTT.S214211 PMC6735535 31564917

[225] Woroniecka K Fecci PE. 4-1bb Agonism as a Strategy to License Immune Checkpoint Blockade in Glioblastoma. Oncoscience (2020) 7:34–5. doi: 10.18632/oncoscience.505 PMC7343573 32676514

[226] Woroniecka KI Rhodin KE Dechant C Cui X Chongsathidkiet P Wilkinson D. 4-1bb Agonism Averts TIL Exhaustion and Licenses PD-1 Blockade in Glioblastoma and Other Intracranial Cancers. Clin Cancer Res (2020) 26:1349–58. doi: 10.1158/1078-0432.CCR-19-1068 PMC7073290 31871298

[227] Croft M So T Duan W Soroosh P. The Significance of OX40 and OX40L to T-Cell Biology and Immune Disease. Immunol Rev (2009) 229:173–91. doi: 10.1111/j.1600-065X.2009.00766.x PMC2729757 19426222

[228] Cao JX Wang H Gao WJ You J Wu LH Wang ZX. The Incidence of Cytokine Release Syndrome and Neurotoxicity of CD19 Chimeric Antigen Receptor-T Cell Therapy in the Patient With Acute Lymphoblastic Leukemia and Lymphoma. Cytotherapy (2020) 22:214–26. doi: 10.1016/j.jcyt.2020.01.015 32305113

[229] Weinkove R George P Dasyam N McLellan AD. Selecting Costimulatory Domains for Chimeric Antigen Receptors: Functional and Clinical Considerations. Clin Trans Immunol (2019) 8:e1049. doi: 10.1002/cti2.1049 PMC6511336 31110702

[230] Shen SH Woroniecka K Barbour AB Fecci PE Sanchez-Perez L Sampson JH. CAR T Cells and Checkpoint Inhibition for the Treatment of Glioblastoma. Expert Opin Biol Ther (2020) 20:579–91. doi: 10.1080/14712598.2020.1727436 PMC7202971 32027536

[231] Grosser R Cherkassky L Chintala N Adusumilli PS. Combination Immunotherapy With CAR T Cells and Checkpoint Blockade for the Treatment of Solid Tumors. Cancer Cell (2019) 36:471–82. doi: 10.1016/j.ccell.2019.09.006 PMC7171534 31715131

[232] Calderon H Mamonkin M Guedan S. Analysis of CAR-Mediated Tonic Signaling. Methods Mol Biol (2020) 2086:223–36. doi: 10.1007/978-1-0716-0146-4_17 31707680

